# An immune-related signature based on molecular subtypes for predicting the prognosis and immunotherapy efficacy of hepatocellular carcinoma

**DOI:** 10.3389/fimmu.2025.1481366

**Published:** 2025-05-19

**Authors:** Xuhui Sun, Wenlong Jia, Huifang Liang, Henghui Cheng

**Affiliations:** ^1^ Department of Pathology, Tongji Hospital, Tongji Medical College Huazhong University of Science and Technology, Wuhan, Hubei, China; ^2^ Department of Hepatology, Tongji Hospital, Tongji Medical College, Huazhong University of Science and Technology, Wuhan, Hubei, China

**Keywords:** hepatocellular carcinoma, immune-related genes, prognosis, immunotherapy, immunohistochemistry, biomarker

## Abstract

**Background:**

Immunotherapy has emerged as a pivotal therapeutic modality for a multitude of malignancies, notably hepatocellular carcinoma (HCC). This research endeavors to construct a prognostic signature based on immune-related genes between different HCC molecular subtypes, offer guidance for immunotherapy application, and promote its clinical practical application through immunohistochemistry.

**Methods:**

Distinguishing HCC subtypes through Gene set variation analysis and Consensus clustering analysis using the Kyoto Encyclopedia of Genes and Genome (KEGG) pathway. In the TCGA-LIHC cohort, univariate, Lasso, and multivariate Cox regression analyses were applied to construct a novel immune relevant prognostic signature. The Subtype-specific and Immune-Related Prognostic Signatures (SIR-PS) were validated in three prognostic cohorts, one immunotherapy cohort, different HCC cell lines and tissue chips. Further possible mechanism on immunotherapy was explored by miRNA-mRNA interactions and signaling pathway.

**Results:**

This prognostic model, which was based on four critical immune-related genes, *STC2*, *BIRC5*, *EPO* and *GLP1R*, was demonstrated excellent performance in both prognosis and immune response prediction of HCC. Clinical pathological signature, tumor microenvironment and mutation analysis also proved the effective prediction of this model. Spatial transcriptome analysis shows that STC2 and BIRC5 are mainly enriched in liver cancer cells and their mRNA and protein expression levels were greater in higher malignant HCC cell lines than in the lower ones. Further validation on HCC tissue chips of this model also showed good correlation with cancer prognosis. The risk score of each patient demonstrated that the SIR-PS exhibited excellent 1 and 3-year survival prediction performance.

**Conclusions:**

Our analysis demonstrates that the SIR-PS model serves as a robust prognostic and predictive tool for both the survival outcomes and the response to immunotherapy in hepatocellular carcinoma patients, which may shed light on promoting the individualized immunotherapy against hepatocellular carcinoma.

## 1 Introduction

According to the 2020 Global Cancer Statistics, liver cancer is the sixth most common human malignancy and the third leading cause of cancer related deaths worldwide, in which liver hepatocellular carcinoma (HCC) accounts for the vast majority (75%-85%) (1). Characterized by nonspecific symptoms and pronounced heterogeneity in the early phases, HCC is often diagnosed at advanced stages, precluding the possibility of curative surgery for the majority of patients (2). Even with the emergence of immunotherapeutic and targeted therapies, the 5-year survival rate for HCC patients remains below 20% (3). The prognosis of patients with HCC is highly variable, which is attributable to its inherent heterogeneity (4). Consequently, there is a pressing need for a novel signature that leverages tumor heterogeneity to predict patient prognosis and select immunotherapy candidates for precision medicine.

Cancer immunotherapy activates the immune system to induce the death of cancer cells (5). The tumor microenvironment (TME), which includes immune cells, stromal cells, the extracellular matrix, and peripheral blood vessels, significantly influences tumor proliferation, metabolic processes, and metastatic potential (6). What’s more, TME plays a vital role in response to cancer immunotherapy in patients with HCC. Amidst the rapid advancements in immunotherapy, its role in HCC treatment is increasingly pivotal.

High-throughput transcriptome sequencing has been widely used in recent years for both clinical and research purposes. However, stringent requirements, intricate procedures and elevated costs impeded its widespread adoption. Immunohistochemistry (IHC) offers a practical and economical alternative for determining protein expression via antibody-mediated staining. Currently, the majority of studies rely on RNA-Seq data for prognostic assessments, whereas models utilizing IHC are limited. If gene-guided predictions can ultimately be validated and applicated through IHC, that will provide a more convenient and cost-effective option.

**Figure 1:**
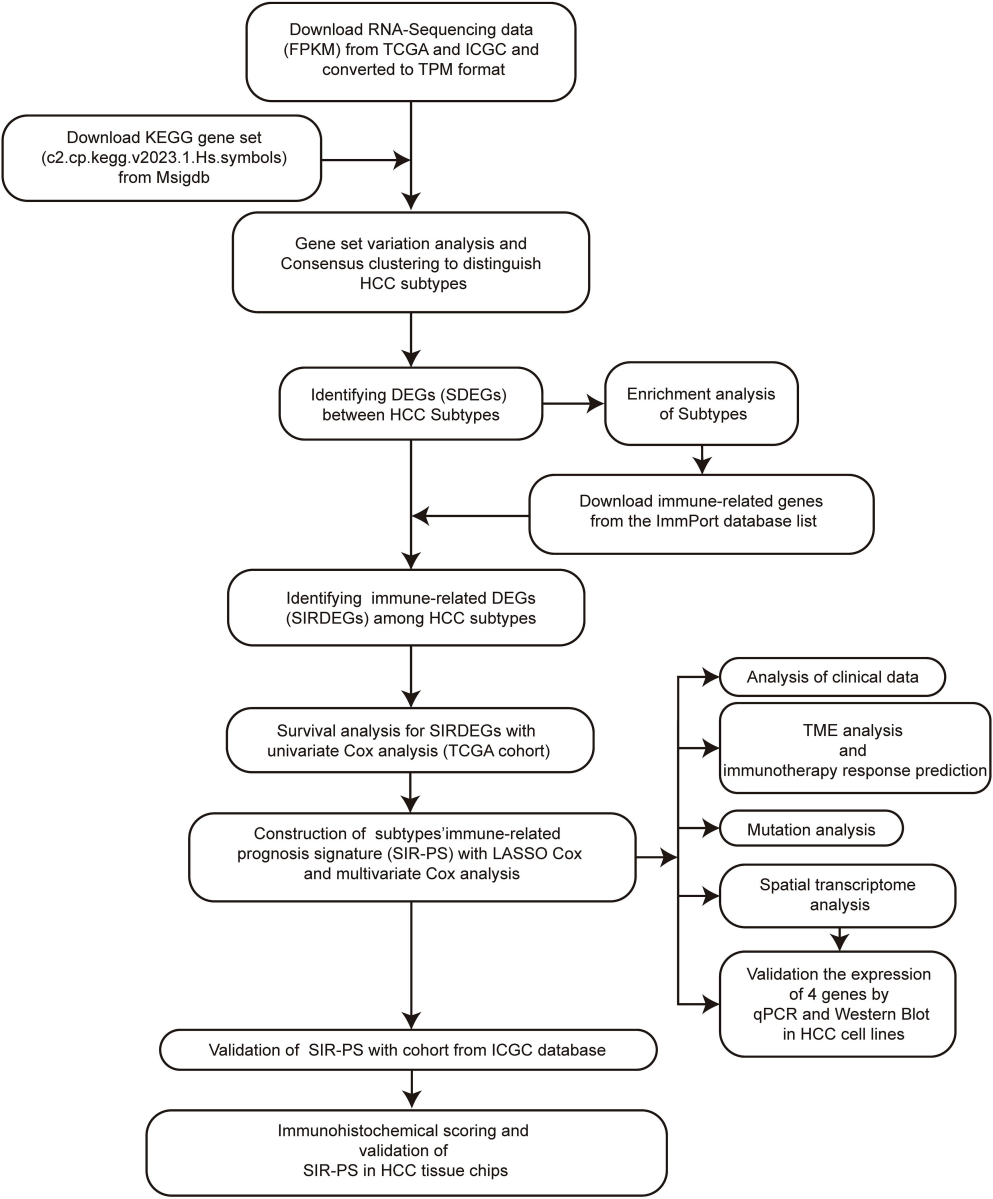
Flow diagram of the analysis procedure: data collection, preprocessing, analysis and validation.

Therefore, in this study, we established a prognostic model based on HCC subtypes and immune related genes. This model was also proofed by the immunohistochemical score to facilitate clinical prognosis and treatment. **Figure 1** illustrates the methodological steps undertaken in this study. The findings might provide insights for future IHC-based studies and contribute to advanced individualized immune therapies for HCC.

## 2 Materials and methods

### 2.1 Data resources

This investigation procured RNA -Seq, clinical, and SNP data from HCC patients through the TCGA (https://portal.gdc.cancer.gov/) and ICGC (https://dcc.icgc.org/) databases, with the exclusion of subjects lacking complete overall survival (OS) data or having survival durations of less than 30 days. The GSE54236 dataset and GSE202069 dataset, sourced from the GEO (https://www.ncbi.nlm.nih.gov/geo/) database, were incorporated into this analysis. The complete TCGA-LIHC cohort served as the training set, while the ICGC- LIRI-JP, GSE54236 and GSE202069 cohorts were utilized as validation datasets.

### 2.2 Gene set variation analysis and consensus clustering

The GSVA algorithm, implemented in the “GSVA” package (7), was employed to derive the relative enrichment scores for the entirety of Kyoto Encyclopedia of Genes and Genome (KEGG) pathways that referenced from the MSigDB (c2.cp.kegg.v2023.1.Hs.symbols) for the comprehensive TCGA cohort (8, 9).

Unsupervised hierarchical clustering of all HCC patients from the TCGA cohort was conducted using the “ConsensusClusterPlus” package (10) to discern distinct HCC subtypes. This procedure entailed 1000 iterations, sampling 80% of the dataset per iteration, to ascertain the stability and reliability of the resulting clusters. The optimal cluster number was determined through the application of the proportion of ambiguous clustering algorithm (11, 12).

### 2.3 Differential and enrichment analysis of the subtypes

Using the “DESeq2” software package for differential analysis, screening differential expressed genes between the two subtypes (SDEGs) based on adjusted P value<0.05 and absolute value of logFC>1 as criteria (13, 14). Utilizing the “clusterProfiler” R package (15), we performed enrichment analysis on above differential genes using gene sets from diverse databases, including OMIM disease gene set, OMIM expanded gene set, ClinVar 2019 gene set, and Rare Diseases GeneRIF Gene Lists gene sets (16–18).

### 2.4 Identification of immune-related differentially expressed genes among HCC subtypes (SIRDEGs)

Immune-related genes (IRGs) were identified from the Immunology Database and Analysis Portal (ImmPort, https://immport.niaid.nih.gov/). Intersection of SDEGs and IRGs to obtain SIRDEGs.

### 2.5 Construction and validation of a prognostic signature based on the SIRDEGs

The TCGA-LIHC Cohort was utilized as the training set for model development. Validation was conducted using the ICGC-LIRI Cohort, the GSE54236 and GSE202069 datasets. Univariate and least absolute shrinkage and selection operator (LASSO) Cox regression analyses were performed using the “survival” and “glmnet” packages to identify the modeling genes. Subtype-specific and Immune-Related Prognostic Signatures, designated the SIR-PS, were identified through multivariate Cox regression. The computational formula for SIR-PS is given by SIR-PS = $\sum_{i}^{n}coef_{i}*mRNA_{i}$ . The R packages “survivalROC” and “survminer” were used to generate time-dependent receiver operating characteristic curves (t-ROC) and Kaplan–Meier survival curves, respectively. The samples were stratified into high-risk and low-risk groups based on the median risk score derived from the TCGA-LIHC cohort. The associations between the SIR-PS and clinicopathological parameters were assessed using the chi-square test and graphically depicted using the “ComplexHeatmap” package (19). Significant clinical parameters were further represented through a stacked bar plot.

### 2.6 Exploration of the tumor immune microenvironment and immunotherapy response

This study utilized the CIBERSORT algorithms for a quantitative assessment of immune cell infiltration, thereby elucidating immunological variations across different groups. Additionally, we scrutinized the expression profiles of immune checkpoint molecules, conducting a comparative analysis to delineate the distinctions between the high-risk and low-risk groups. Furthermore, we leveraged the HCC Immunotherapy Cohort (RNA-Seq data from Li et al.’s study) to substantiate the predictive efficacy of the SIR-PS in forecasting responses to immunotherapy (20).

### 2.7 Mutation analysis

The mutational data of patients in the TCGA-LIHC cohort were obtained from the TCGA database. The “maftools” R package was utilized to evaluate the mutational landscape and compare the mutational spectra between high-risk and low-risk groups of HCC patients (21).

### 2.8 Spatial transcriptome analysis

The spatial transcriptomics data were obtained from Liu et al.’s study (22). According to the authors’ provided data, we calculated the model score for each cell using SIR-PS. We then used the Seurat package to visualize cell types and their corresponding scores.

### 2.9 Quantitative real-time reverse transcriptase polymerase chain reaction in cell lines

Hep3B, Huh7, MHCC-97H (97H), and SNU-449 cell lines (ATCC Cell Bank, United States) were cultured to verify the expression levels of these signature genes. Total RNA was isolated from the aforementioned cell lines utilizing FreeZol Reagent (Vazyme, China) followed by the synthesis of cDNA using a reverse transcription kit (Vazyme, China). qPCR was conducted with SYBR Green Mix (Q711, Vazyme) and a C1000 thermal cycler from Bio-Rad (Hercules, CA). The sequences of the primers used for the signature genes are detailed in **Table 1**. The relative expression levels were normalized to those of the housekeeping gene *GAPDH*.

**Table 1:** The sequences of the primers used in qPCR.

Gene name	Forward primer sequence	Reverse primer sequence
GAPDH	TCCAAAATCAAGTGGGGCGA	TGATGACCCTTTTGGCTCCC
STC2	TGAAATGTAAGGCCCACGCT	ACTGTTCGTCTTCCCACTCG
BIRC5	TCAAGGACCACCGCATCTCT	CCAAGTCTGGCTCGTTCTCA
EPO	AGGCCGAGAATATCACGACG	CAGACTTCTACGGCCTGCTG
GLP1R	AGTCCAAGCGAGGGGAAAGA	GAGGCGATAACCAGAGCAGAG

### 2.10 Western blotting

Cellular lysates were prepared using RIPA lysis buffer. Equal amounts of proteins were subjected to SDS–PAGE and then transferred to polyvinylidene fluoride membranes. The membranes were blocked with a protein-free rapid blocking solution (PS108P, Epizyme) for 20 minutes to prevent nonspecific antibody binding. Primary antibodies (10314, 10508, 26196 from Proteintech, A5663 from ABclonal) were diluted according to the manufacturer’s instructions and incubated at 4°C for 12 hours to allow for antibody-antigen binding. After washing with Tris-Buffered Saline with Tween, secondary antibodies (SA00001 from Proteintech) were applied and incubated for 1 h at room temperature to facilitate signal detection. After washing, the immunoreactive bands on the membranes were visualized using an enhanced chemiluminescence chromogenic substrate.

### 2.11 Validation of SIR-PS in HCC tissue chips

Two HCC tissue chips were obtained from the Department of Liver Surgery at Tongji Hospital, Tongji Medical College, Huazhong University of Science and Technology. Patients with incomplete clinical data or tissue loss excluded from the analysis. The immunohistochemistry staining was performed as described previously (23). The slides were incubated with primary antibodies (anti-*STC2* ab255610, Abcam, anti-*GLP1R* 26196, Proteintech, anti-*EPO* ZRB1366, Sigma, and anti-*BIRC5* ZA0530, ZSGB-BIO). Semiquantitative scores were assigned according to the staining intensity and the proportion of positively stained cells, with the following categories and corresponding scores: no staining (0), light yellow (1), medium yellow (2), dark yellow (3), and heavy yellow (4); multiple with the corresponding positive percentage of stained cells relative to the total number of cells > The composite score for each specimen was calculated as the sum of the products of the staining intensity and the percentage of positively stained cells. Immunohistochemical staining was independently evaluated by two pathologists in a double-blinded manner via microscopy. The HCC tissue chip cohort was stratified into High-risk and Low-risk groups utilizing “surv_cutpoint” from the “survminer” package. The prognostic predictive efficacy of the SIR-PS was confirmed using Kaplan–Meier analysis and t-ROC curves. In the HCC tissue chip cohort, use the “compactGroups” package to generate a three line table to statistically analyze the distribution of clinical pathologica l parameters between different groups for each indicator (24).

### 2.12 Exploring the potential mechanisms of SIR-PS regulating immunotherapy efficacy

Analyze the relationship between modeling genes and PDL1 expression in the TCGA-LIHC cohort. Based on the above results, miRNAs targeting PDL1 and modeling genes correlated with PDL1 expression that have been experimentally validated in the TarBase database were screened using the MultiMiR package. Take the intersection of the miRNA results of the modeling genes mentioned above with the miRNAs targeting PDL1.

### 2.13 Statistical analysis

All the statistical tests and bioinformatics analyses were performed with R software, version 4.0.1. The Wilcoxon rank sum test, Pearson chi-square test, t test and log-sum test were included. P <0.05 was considered to indicate statistical significance.

## 3 Results

### 3.1 Identification and enrichment analysis of subtypes based on KEGG pathway in HCC

**Figure 2:**
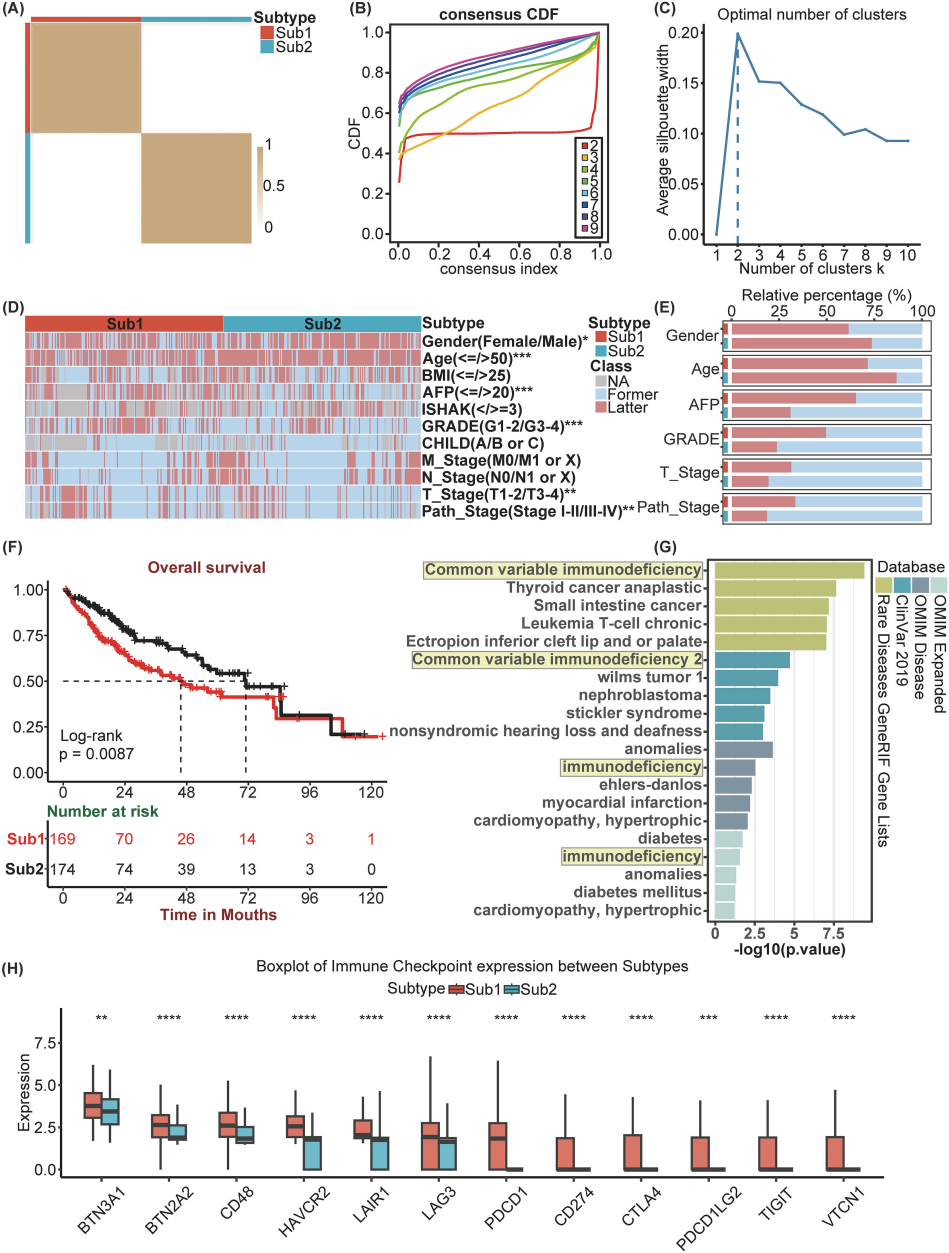
Identification and differential analysis of HCC Subtypes based on KEGG pathways. **(A)** Heatmap of sample clustering at consensus k=2. **(B)** Consensus clustering CDF for k= 2 to 9. **(C)** The Average Silhouette width Plot. **(D)** Heatmap of and **(E)** Stacked bar chart of multiple clinicopathological features between Subtypes. **(F)** Kaplan-Meier survival plots between Subtypes for Overall Survival (0S). **(G)** Enrichment analysis of diseases associated with Sub1 enrichment genes. **(H)** Immune Checkpoint genes’ expression between Subtypes. *p<0.05, **p<0.01, ***p<0.001, ****p<0.0001.

Utilizing the enrichment scores of KEGG gene sets based on the GSVA algorithm, we conducted unsupervised hierarchical clustering to classify the samples into two distinct subtypes, which were validated by the examination of the cluster heatmap, the consensus CDF plot, the average silhouette width, and the Proportion of Ambiguous Clustering algorithm (**Figures 2A-C**). Consequently, the patients of TCGA-LIHC cohort was stratified into two distinct molecular subtypes (**Supplementary Table 1**). Subsequently, a comparative analysis of the clinical factors across different subtypes was conducted, employing heatmap for visualization (**Figure 2D**). Additionally, stacked bar charts were utilized to highlight factors exhibiting significant inter-subtype disparities (**Figure 2E**). Compared to Sub2, Sub1 is characterized by elevated levels of AFP, a higher GRADE, advanced path stage and T stage, a greater proportion of female patients, and a lower median age. As indicated by the Kaplan–Meier analysis, patients classified into Sub2 exhibited a more favorable prognosis than those classified into Sub1 (**Figure 2F**). In light of the observed disparities in survival outcomes, we employed the “DESeq2” package to perform a differential analysis between the two identified subtypes. Employing LogFC>1 as the criterion, Sub1 and Sub2 were found to harbor 2284 and 751 differentially expressed genes, respectively. The enrichment analysis conducted on Sub1 disclosed that in the gene sets of the four databases, OMIM disease, OMIM Expanded, ClinVar2019, and Rare Disease GeneRIF GeneLists, the genes enriched by Sub1 are unanimously associated with immunodeficiency diseases (**Figure 2G**). Additionally, an analysis of immune checkpoint expression levels among the subtypes was performed. This analysis indicated that the expression levels of immune checkpoint genes in Sub1 were, on the whole, markedly elevated compared to those in Sub2 (**Figure 2H**).

### 3.2 Development and validation of the SIR-PS

**Figure 3:**
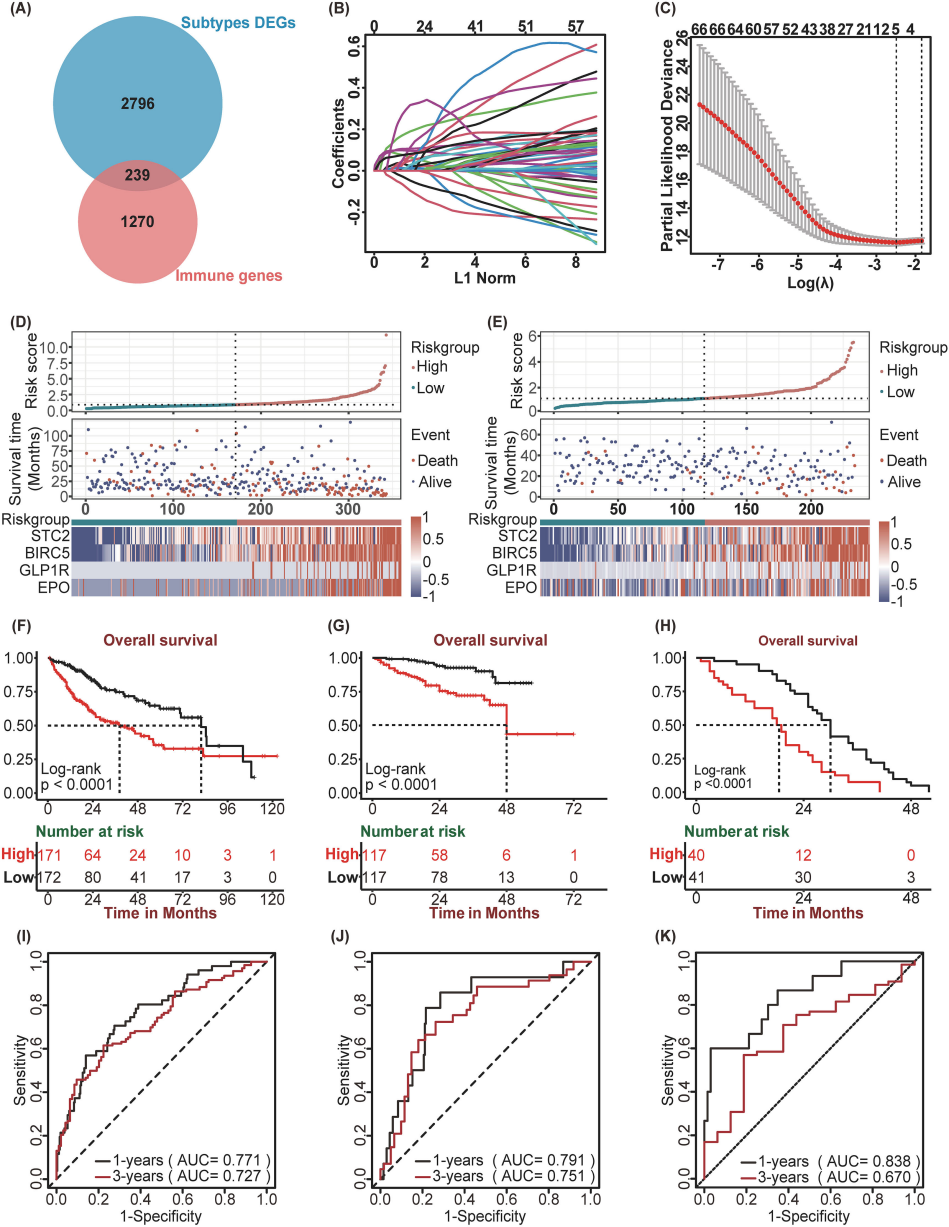
Construction and Validation of SIR-PS. **(A)** Venn plot showed 239 immune-related DEGs among subtypes. **(B)** LASSO coefficient profiles of 67 prognostic genes of HCC. **(C)** 10-fold cross validated lasso regression identified five prognostic genes with minimal λ. **(D, E)** Riskscore distribution, survival status, and expression of four SIR-PS signature genes of patients in the Low-risk and High-risk group of TCGA Cohort and ICGC Cohort, respectively. **(F–H)** Kaplan-Meier survival plots of High-risk and Low-risk group for Overall Survival in the TCGA Cohort, the ICGC Cohort and the GSE54236 Cohort. **(I–K)** Time-dependent ROC curves of SIR-PS for Overall Survival in the TCGA Cohort, the ICGC Cohort and the GSE54236 Cohort.

In the TCGA-LIHC cohort we obtained 3035 SDEGs (**Supplementary Figure 1**). Then, SDEGs were intersected with 1,509 immune-related genes obtained from the ImmPort database, yielding a total of 239 immune-related SDEGs (SIRDEGs) (**Figure 3A**). Univariate Cox regression analysis revealed 67 SIRDEGs with significant prognostic potential (**Supplementary Table 2**). Then, LASSO regression analysis was performed, and five SIRDEGs were further identified for modeling (**Figures 3B, C**). Four genes, *STC2*, *BIRC5*, *EPO*, and *GLP1R*, were identified for their substantial influence on the prognostic model. The group with high expression levels of these genes exhibited a markedly poorer prognosis than the group with low expression (**Supplementary Figures 2A-D**). These genes were subsequently utilized to construct a prognostic model (called SIR-PS) through multivariate Cox regression analysis, resulting in the following risk score formula: risk score = (*STC2* × 0.22344) + (*BIRC5* × 0.19238) + (*EPO* × 0.11058) + (*GLP1R* × 0.24472). The four-gene model demonstrated a prediction performance closely comparable to that of the five-gene model (**Supplementary Figures 2E, F**). Subsequently, 343 TCGA-LIHC patients were stratified into low-risk and high-risk groups based on the median risk score. In addition, we plotted ensemble plots of survival status and four signature gene expression profiles as the risk score increased (**Figures 3D, E**). There was a progressive increase in both mortality rates and the expression levels of the four signature genes concomitant with increasing risk scores. Kaplan–Meier analysis revealed that patients in the high-risk group experienced a more adverse clinical prognosis than did those in the low-risk group (**Figure 3F**). The AUC value at 1 and 3 years were 0.771 and 0.727 respectively, which is indicative of the model’s robust predictive capability (**Figure 3I**). Leveraging the SIR-PS, we computed individual risk scores for all HCC patients within the ICGC cohort. These scores were then stratified to distinguish between high-risk and low-risk groups based on the median value of the risk scores. Consistent with the findings in the TCGA-LIHC cohort, the Kaplan–Meier analysis demonstrated that the OS of patients in the high-risk group was significantly inferior to that of patients in the low-risk group in ICGC-LIRI-JP cohort (**Figure 3G**). The AUCs for the ICGC-LIRI-JP cohort at 1 and 3 years were 0.791 and 0.751, respectively (**Figure 3J**). In the GSE54236 cohort and the GSE202069 cohort, the Kaplan–Meier analysis revealed that patients in the high-risk group experienced significantly shorter OS than those in the low-risk group (p<0.0001 and p=0.08, respectively) (**Figure 3H**, **Supplementary Figure 3A**). The AUCs for the GSE54236 cohort and the GSE202069 cohort at the 1-year were 0.838 and 0.818, respectively, while at the 3-year they were 0.67 and 0.866, respectively (**Figure 3K**, **Supplementary Figure 3B**).

In addition, we compared the three-year survival prediction performance of SIR-PS with nine other prognostic models in four datasets (25–33). The results showed that the AUC value of SIR-PS had the best predicted performance in these datasets (**Supplementary Figure 3C**). Taking the average AUC value at 1 and 3 year of four datasets, the AUC value of SIR-PS ranked the second and first respectively, which also proofed its comprehensive prediction value.

### 3.3 Exploration of the clinical significance and tumor microenvironment of the SIR-PS

**Figure 4:**
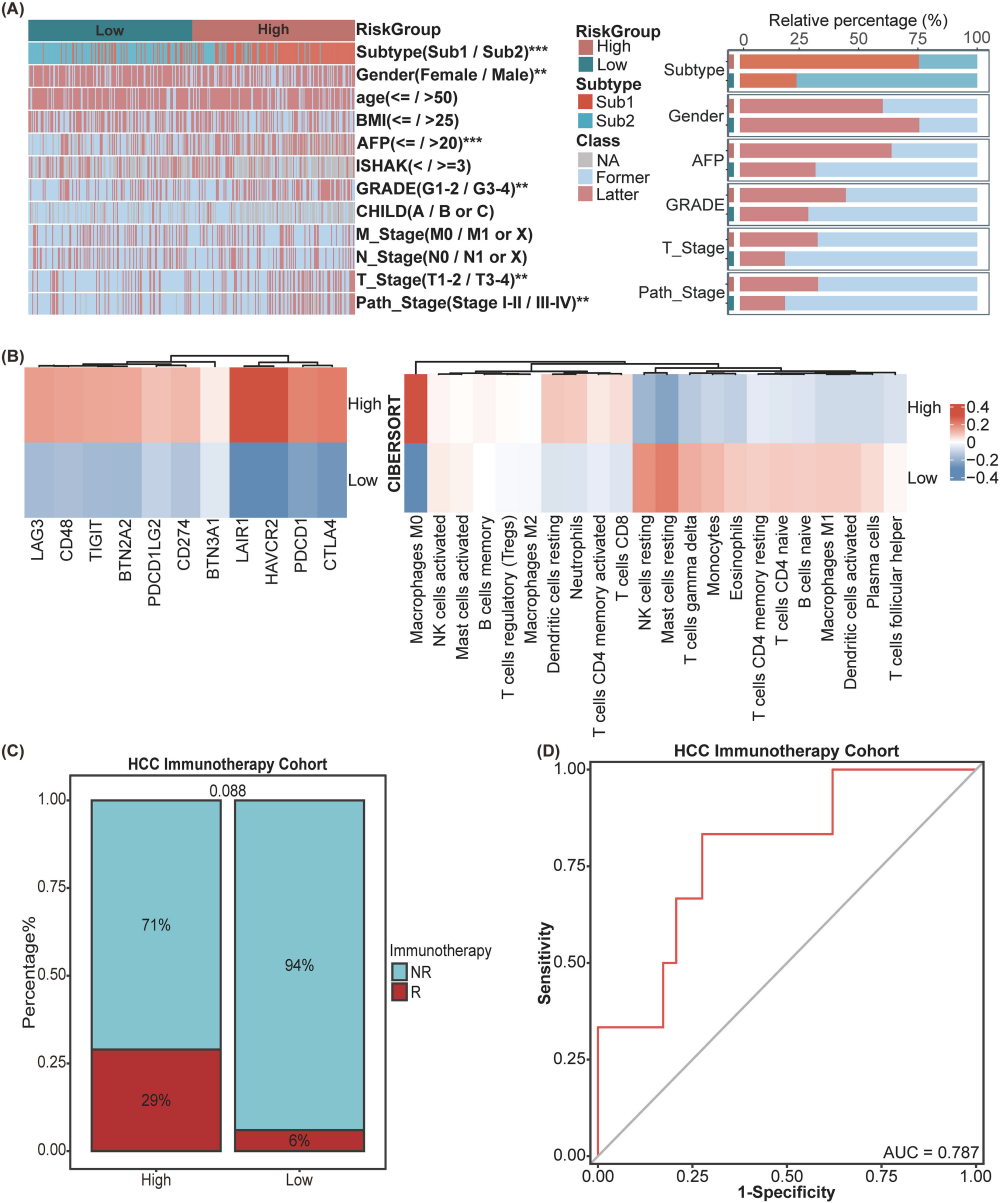
Exploration of clinical significance and tumor microenvironment of SIR-PS in the TCGA Cohort. **(A)** Heatmap and Stacked bar chart of multiple clinicopathological features between High-risk and Low-risk group of SIR-PS. **(B)** Heatmap of Immune Checkpoint expression and CIBERSORT result between High-risk and Low-risk group of SIR-PS. **(C)** Stacked bar chart of immunotherapy response between High-risk and Low-risk group of SIR-PS in our HCC Immunotherapy Cohort. **(D)** Diagnostic ROC plot of SIR-PS predicting response to immunotherapy. **p<0.01, ***p<0.001.

To investigate the association between the SIR-PS and a range of clinicopathological characteristics, the correlation analysis was conducted and revealed significant associations between the risk groups and various HCC features (**Figure 4A**). The high-risk group exhibited increased levels of AFP, a greater percentage of patients within Sub1 and female patients, more advanced GRADE, and higher pathological stage and T stage than did the low-risk group (**Figure 4A**). Subsequently, leveraging the CIBERSORT algorithm, we quantified the infiltration levels of various immune cells across samples and delineated the comparative immune landscape between the high-risk and low-risk groups within the TCGA cohort. The analysis delineated that the high-risk group was distinguished by an enhanced infiltration of B cells memory, T cells regulatory, Dendritic cells resting, Neutrophils, T cells CD4 memory activated and T cells CD8 and a diminished presence of NK cells activated, Mast cells activated and resting, Macrophages M1, Dendritic cells activated (**Figure 4B**). Further analysis of immune checkpoint gene expression between risk groups within the TCGA database revealed that the high-risk group displayed elevated expression levels for the majority of these genes, in contrast to the low-risk group (**Figure 4B**). Concurrently, we assessed the predictive efficacy of SIR-PS concerning the response to immunotherapy within the HCC Immunotherapy Cohort, with results indicating a higher response rate among patients in the high-risk group as per the median risk score (**Figure 4C**). The t-ROC curve analysis revealed that the AUC value for predicting treatment responsiveness based on the risk score was 0.787 (**Figure 4D**).

### 3.4 Mutation landscape analysis of SIR-PS

**Figure 5:**
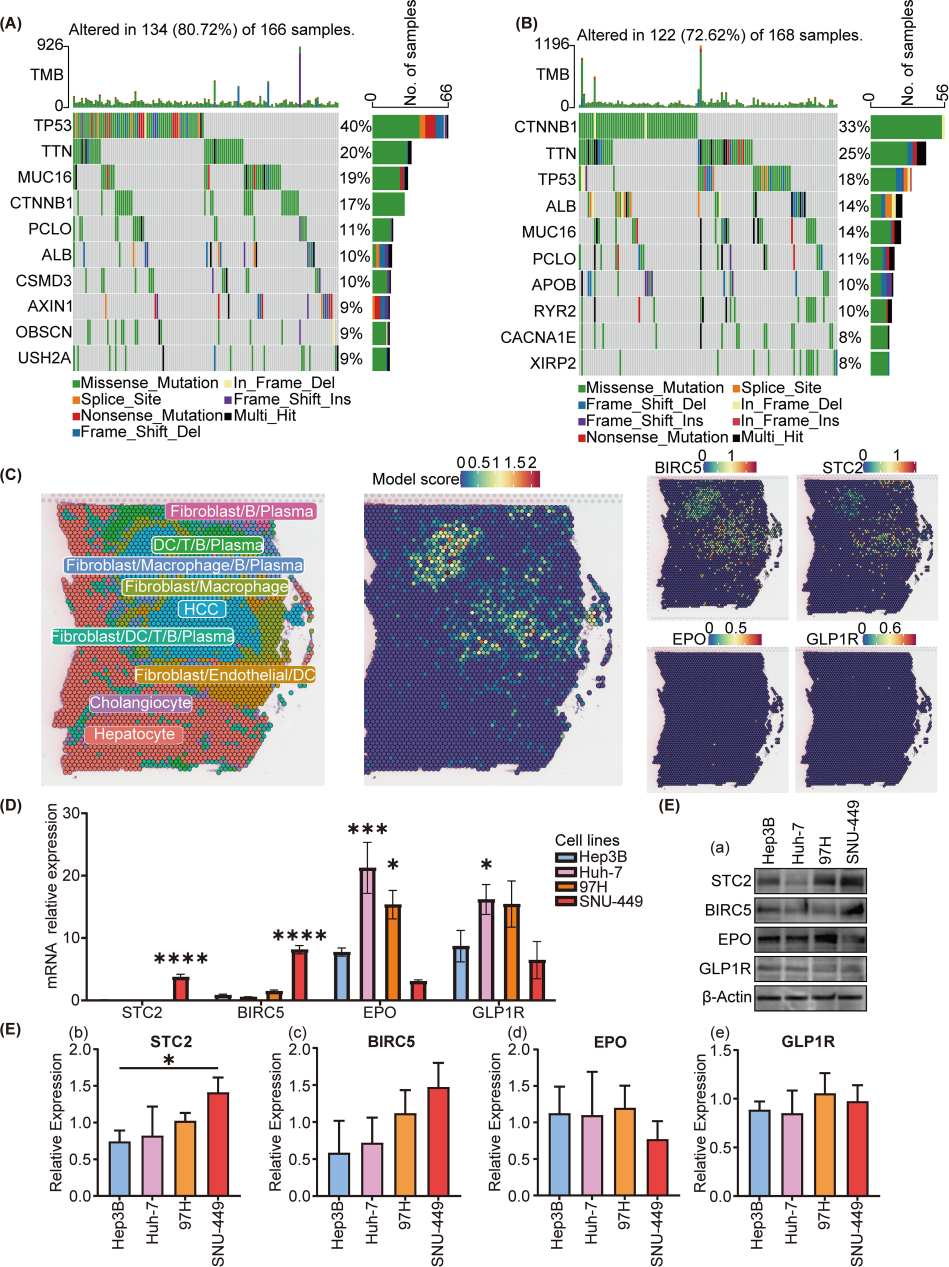
Mutational and spatial transcriptome analysis of SIR-PS risk groups and cell experiment of different cell lines. **(A, B)** Oncoplot analysis of the high-risk and low-risk group, respectively. **(C)** Spatial expression pattern of SIR-PS (including BIRC5, STC2, EPO and GLP1R). **(D)** qPCR and **(E)** Western Blotting result of Hep3B, Huh7, 97H and SNU-449 (compare with Hep3B cell lines). *p<0.05, ***p<0.001, ****p<0.0001.

Initially, we scrutinized the 10 genes exhibiting the highest mutation frequencies within the low-risk and high-risk group. Oncoplots revealed that within the TCGA database, the genes exhibiting the highest mutation frequencies in the high-risk and low-risk groups were TP53, with a 40% mutation frequency, and CTNNB1, with a 33% mutation frequency, respectively (**Figures 5A, B**).

### 3.5 Spatial transcriptome analysis of SIR-PS

To determine the cell types in which our model is active, we analyzed spatial transcriptomics data from HCC patients. Our analysis revealed that the riskscores highest in HCC cells, indicating that the SIR-PS’s riskscore in patients is predominantly determined by its riskscore in these cancer cells (**Figure 5C**). Concurrently, *STC2* and *BIRC5* exhibit predominant expression within HCC cells.

### 3.6 qPCR and Western blotting in HCC cell lines

In light of the spatial transcriptome analysis findings, we chose HCC cell lines, including SNU-449, 97H, Hep3B and Huh7, to conduct cellular-level validation studies. The SNU-449 and 97H cell lines exhibited a greater degree of malignancy or transfer ability than the Hep3B and Huh7 cell lines, which commonly means a worse prognosis (34, 35). No matter in the qPCR or the western blotting detection, the expression levels of *STC2* and *BIRC5* were higher in the SNU-449 and 97H cell lines than in the Huh7 and Hep3B cell lines (**Figures 5D, E**), which were in accordance with their malignancies. However *EPO* and *GLP1R* showed not obvious trends in the mRNA and protein levels.

### 3.7 Validation of the SIR-PS based on iHC staining of the HCC tissue chips

**Figure 6:**
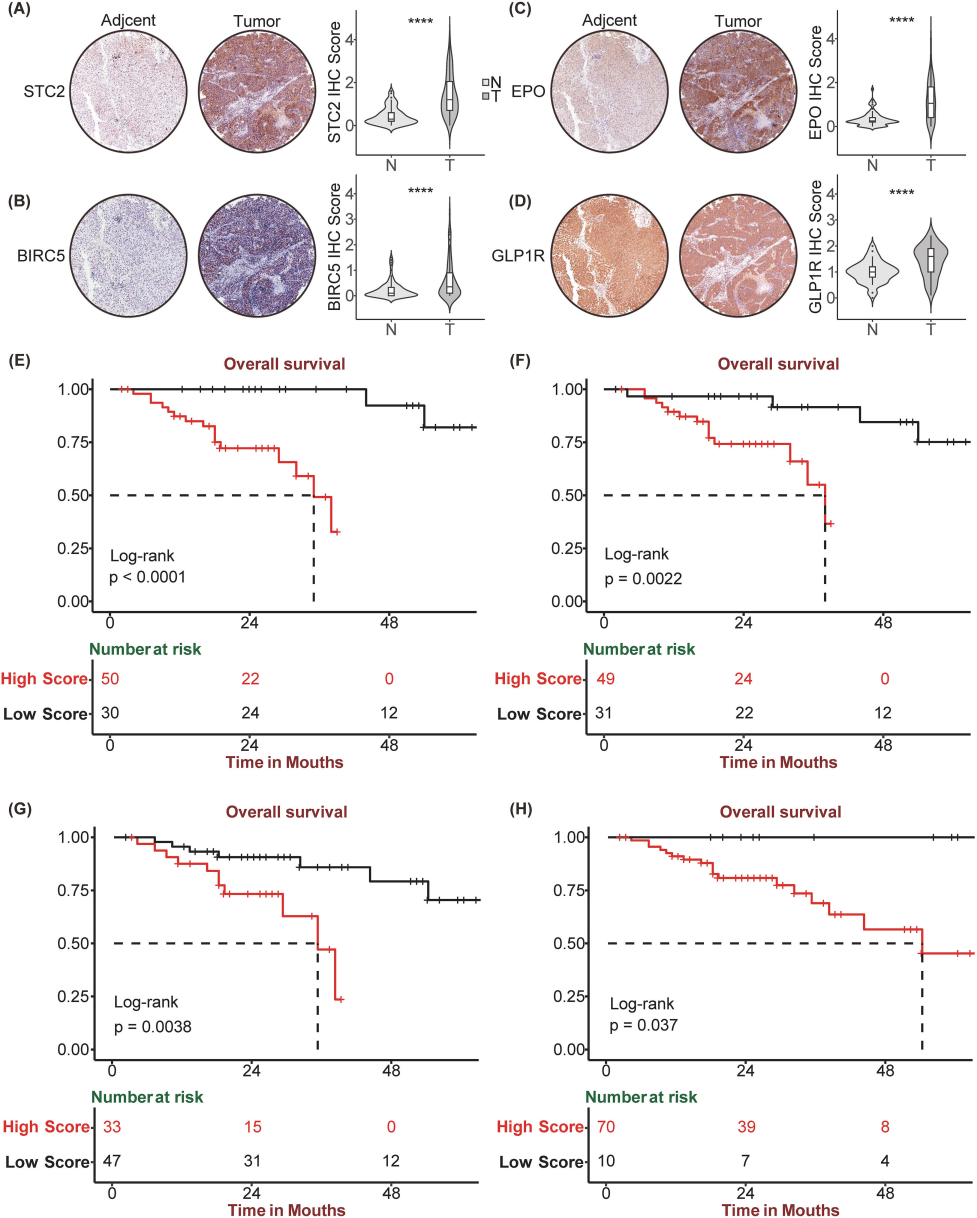
Immunohistochemistry staining and corresponding Kaplan Meier analysis of STC2, BIRC5, EPO, GLP1R. Tumor and paired Normal tissue IHC staining of HCC tissue chip by STC2 **(A)**, BIRC5 **(B)**, EPO **(C)**, GLP1R **(D)**. Kaplan-Meier curve between high and low expression of STC2 **(E)**, BIRC5 **(F)**, EPO **(G)**, GLP1R **(H)** in HCC tissue chip Cohort, respectively. ****p<0.0001.

**Figure 7:**
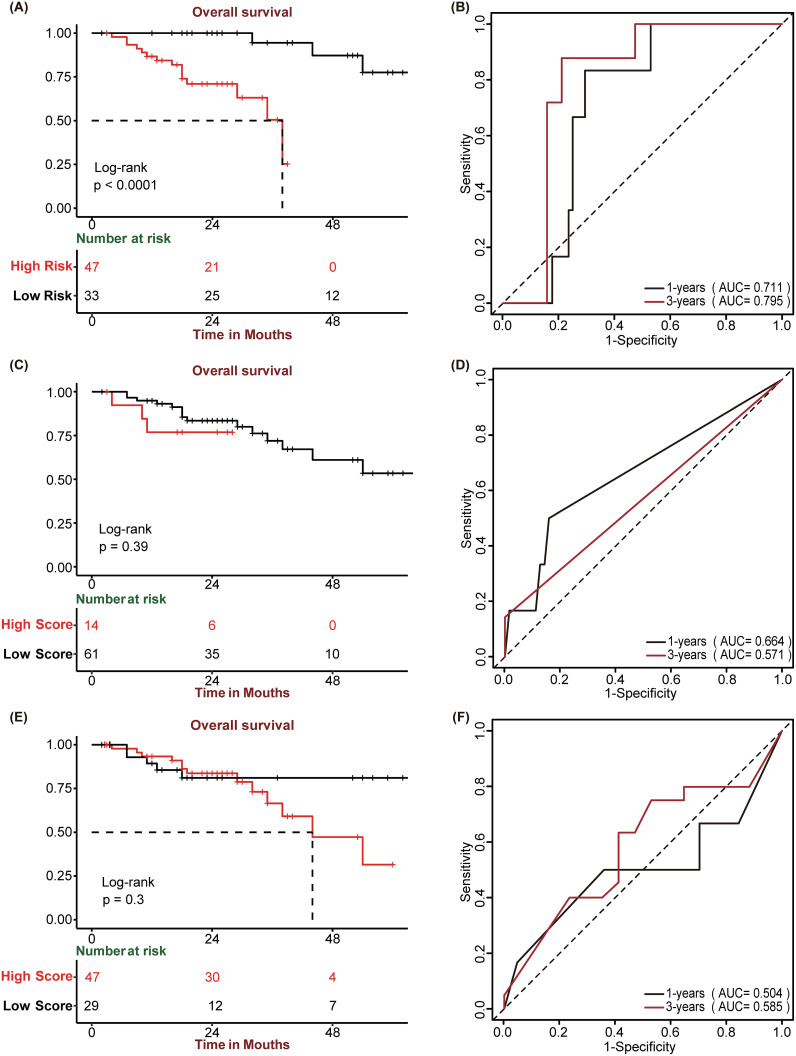
Prognostic performance of SIR-PS, GPC3, and CK19. **(A, C, E)** Kaplan-Meier survival plots of SIR-PS risk group, CK19 and GPC3 expression group for Overall Survival in the HCC tissue chip Cohort. **(B, D, F)** Time-dependent ROC curves of SIR-PS, CK19 and GPC3 for Overall Survival in the HCC tissue chip Cohort.

Owing to the remarkable prognostic potential of the four signature genes, we conducted IHC staining on tissue chips sourced from HCC patients and subsequently scored the expression of these genes. Post-IHC staining revealed that *STC2*, *BIRC5*, *EPO*, and *GLP1R* exhibited increased expression in HCC tissues relative to normal controls (**Figures 6A–D**). Utilizing the “surv_cutpoint” function from the “survminer” package, the IHC scores for each gene were stratified into high-IHC and low-IHC groups. Kaplan–Meier analysis demonstrated that Patients in the high-IHC group for *STC2*, *BIRC5*, *EPO* and *GLP1R* exhibited a markedly poorer prognosis than did those in the low-IHC group (**Figures 6E-H**). Subsequently, we calculated the riskscore of each patient of HCC tissue chips using SIR-PS based on the IHC score of four genes. The riskscores of patients were subsequently categorized into high-risk and low-risk group using “surv_cutpoint” function of “survminer” package. Based on the calculated risk scores, patient stratification into high-risk and low-risk groups was determined using a cutoff value of 0.6115285. Kaplan–Meier analysis indicated that across the entire HCC tissue chip cohort, the high-risk group had a significantly worse prognosis than did the low-risk group (**Figure 7A**). ROC curve analysis revealed that the AUC value for the entire HCC tissue chip cohort at the 1 and 3-year was 0.711 and 0.795, respectively (**Figure 7B**). Furthermore, given that *GPC3* and *CK19* are commonly used prognostic markers in clinical liver cancer diagnostics, we also conducted IHC staining for these markers on HCC tissue chips and scored them accordingly. Subsequent to their score, these two prognostic indicators were evaluated independently to predict patient outcomes. Kaplan-Meier analysis revealed no significant survival disparity between the high-IHC and low-IHC groups for *CK19* and *GPC3* across the entire HCC tissue chips cohort (**Figures 7C, E**). Correspondingly, the 1-year AUC values of their respective t-ROC curves were 0.664 and 0.504, while the 3-year AUC values were 0.571 and 0.585, respectively (**Figures 7D, F**).

### 3.8 Exploration of clinical information between high-risk and low-risk group of patients with HCC tissue chips data

A comparative analysis of the clinical characteristics between different groups was conducted. Summary descriptives table of general clinical factors of all patients and riskgroup is shown in **Table 2**, while the different indicators groups are shown in **Supplementary Table 3**. Based on the clinical data and varying classifications of staining and risk groups, we conducted both univariate and multivariate Cox regression analysis (**Table 3**). The results of univariate Cox regression analysis showed that there were significant differences in survival between AST, childpugh, tumor size, vascular invasion, *BIRC5*, *EPO* and risk groups. STC2 and GLP1R cannot be subjected to Cox regression analysis due to the fact that the number of deceased patients in one of the high and low IHC groups is less than 3. Given that risk group are determined by the expression levels of *STC2*, *BIRC5*, *GLP1R*, and *EPO*, we prioritized risk group, and other factors exhibiting significant intergroup survival differences for inclusion in the multivariate Cox regression analysis. In the multivariate Cox regression analysis, the risk group remained the sole significant predictor, with a p-value<0.05.

**Table 2:** Summary descriptives table of all patients and riskgroup in the tissue chips cohort.

	All	IHC Riskgroup		
		Low	High	p.overall
N:	80 (100%)	33 (41.2%)	47 (58.8%)	
Gender:				0.233
Female	16 (20.0%)	4 (12.1%)	12 (25.5%)	
Male	64 (80.0%)	29 (87.9%)	35 (74.5%)	
Age:				0.79
<=50	39 (48.8%)	15 (45.5%)	24 (51.1%)	
>50	41 (51.2%)	18 (54.5%)	23 (48.9%)	
ALT:				1
<=41	61 (76.2%)	25 (75.8%)	36 (76.6%)	
>41	19 (23.8%)	8 (24.2%)	11 (23.4%)	
AST:				0.234
<=40	56 (70.0%)	26 (78.8%)	30 (63.8%)	
>40	24 (30.0%)	7 (21.2%)	17 (36.2%)	
AFP:				1
<=20	20 (25.3%)	8 (24.2%)	12 (26.1%)	
>20	59 (74.7%)	25 (75.8%)	34 (73.9%)	
Child-Pugh:				0.139
A	76 (95.0%)	33 (100%)	43 (91.5%)	
B	4 (5.00%)	0 (0.00%)	4 (8.51%)	
Cirrhosis:				0.707
No	26 (32.5%)	12 (36.4%)	14 (29.8%)	
Yes	54 (67.5%)	21 (63.6%)	33 (70.2%)	
Tumor number:				0.933
1	59 (73.8%)	25 (75.8%)	34 (72.3%)	
>1	21 (26.2%)	8 (24.2%)	13 (27.7%)	
Tumor size:				1
<=5cm	31 (38.8%)	13 (39.4%)	18 (38.3%)	
>5cm	49 (61.3%)	20 (60.6%)	29 (61.7%)	
Vascular invasion:				1
No	66 (82.5%)	27 (81.8%)	39 (83.0%)	
Yes	14 (17.5%)	6 (18.2%)	8 (17.0%)	
Differentiation:				0.389
Moderate or High	54 (67.5%)	20 (60.6%)	34 (72.3%)	
Low or Moderately low	26 (32.5%)	13 (39.4%)	13 (27.7%)	
BCLC.stage:				0.981
A	52 (65.0%)	22 (66.7%)	30 (63.8%)	
B or C	28 (35.0%)	11 (33.3%)	17 (36.2%)	
TNM.stage:				0.475
1 or 2	61 (76.2%)	27 (81.8%)	34 (72.3%)	
3 or 4	19 (23.8%)	6 (18.2%)	13 (27.7%)	

**Table 3:** Cox Univariate and Multivariable regression analysis between cumulative overall survival rate and clinicopathological variables of all patients in the HCC tissue chip.

Variables	Univariate analysis		Multivariable analysis	
	HR (95% CI)	P-value	HR (95% CI)	P-value
Gender (Male/Female)	1.69 (0.385-7.41)	0.487		
Age (>50/<=50)	1.42 (0.55-3.68)	0.467		
ALT (>41/<=41)	1.33 (0.473-3.76)	0.586		
AST (>40/<=40)	4.4 (1.7-11.4)	0.00229	2.32 (0.777-6.9)	0.132
ALP (>130/<=130)	1.8 (0.589-5.48)	0.303		
AFP (>20/<=20)	0.656 (0.245-1.75)	0.401		
ChildPugh (B/A)	8.95 (2.36-34)	0.00129	2.81 (0.679-11.6)	0.154
Cirrhosis (Yes/No)	1.08 (0.403-2.87)	0.884		
Tumornumber (>1/1)	1.6 (0.596-4.3)	0.351		
Tumorsize (>5cm/<=5cm)	3.75 (1.08-13)	0.0371	2.5 (0.663-9.4)	0.176
Vascularinvasion (Yes/No)	3.66 (1.25-10.7)	0.0181	1.95 (0.584-6.52)	0.278
Differentiation (Low or Moderately low/Moderate or High)	1.64 (0.63-4.25)	0.312		
BCLCstage (B or C/A)	2.54 (0.994-6.49)	0.0514		
TNMstage (3 or 4/1 or 2)	2.39 (0.917-6.24)	0.0747		
STC2group (High/Low)	7.45e+08 (0-Inf)	0.997		
BIRC5group (High/Low)	7.99 (1.74-36.8)	0.00763		
GLP1Rgroup (High/Low)	2.71e+08 (0-Inf)	0.998		
EPOgroup (High/Low)	4.41 (1.48-13.1)	0.0076		
RiskGroup (High/Low)	22.8 (2.85-182)	0.0032	23.8 (2.74-207)	0.00405

### 3.9 Exploration of the mechanism by which prognostic models affect immunotherapy

**Figure 8:**
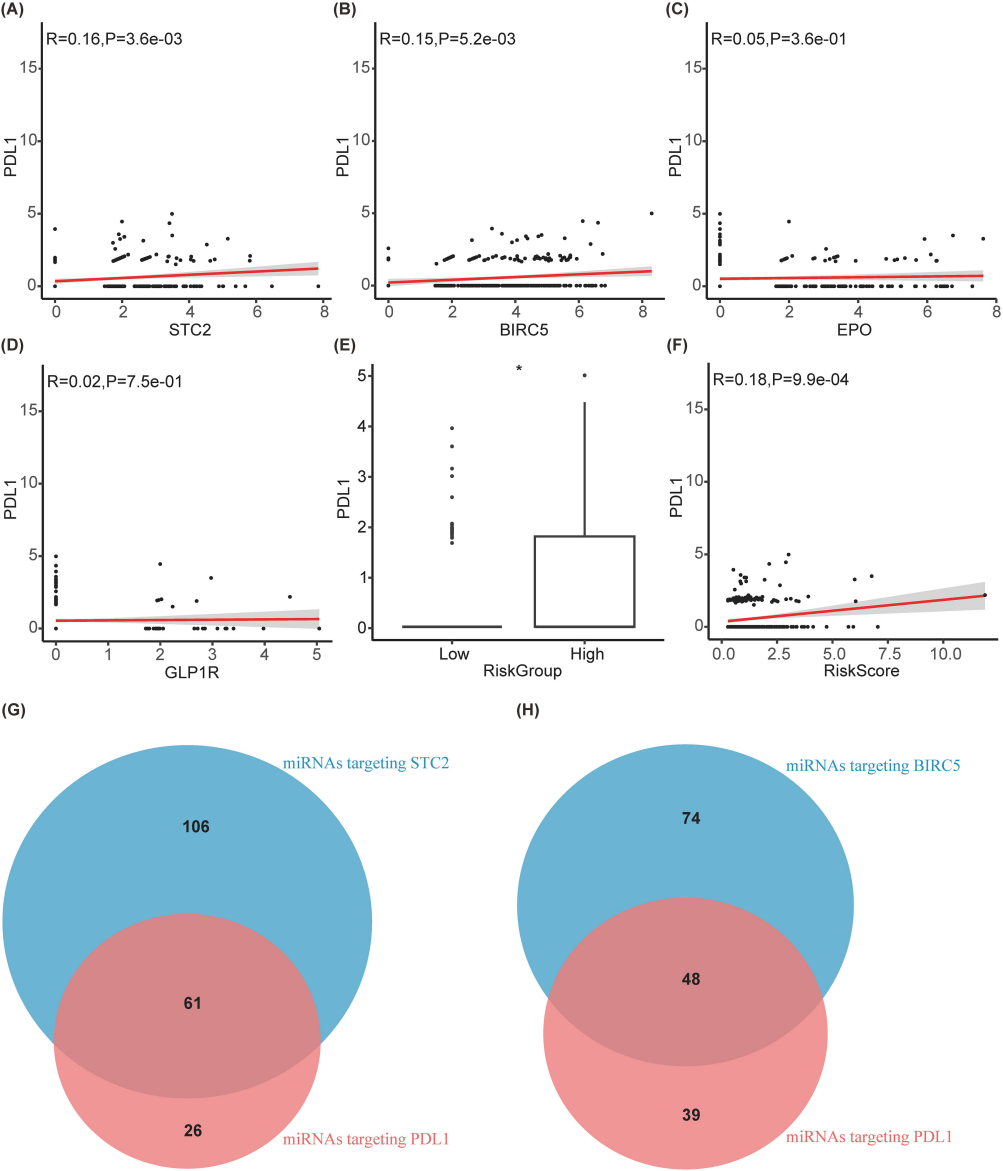
Exploration of the mechanism by which prognostic models affect immunotherapy. **(A–D)** Correlation diagram between PDL1 and STC2, BIRC5, EPO, GLP1R, respectively. **(E)** Boxplot between PDL1 and riskgroup. **(F)** Correlation diagram between PDL1 and riskscore. **(G, H)** Venn diagram of miRNAs targeting PDL1 with targeting STC2 and BIRC5, respectively.

Due to the good predictive effect of risk scores in the liver cancer immunotherapy queue treated with anti-PD1/PDL1, SIR-PS may affect the efficacy of immunotherapy by affecting PDL1 expression. In the TCGA-LIHC cohort, the expression levels and risk scores of STC2 and BIRC5 were positively correlated with the expression level of PDL1 (**Figures 8A, B, E, F**), indicated that both STC2 and BIRC5 can promote the expression of PDL1 on cancer cells, thereby promoting tumor immune escape. However, there is no obvious correlation between EPO or GLP1R and PDL1 (**Figures 8C, D**). Further exploration on the potential mechanism of STC2 and BIRC5 regulating PDL1 was conducted. Since the potential mutual influence of gene expression through miRNAs, the multiMIiR package was used to screen miRNAs in the TarBase database that have been experimentally validated to bind to STC2, BIRC5, and PDL1. There are a total of 87 miRNAs targeting PDL1, with 61 shared miRNAs between PDL1 and STC2, and 48 shared miRNAs between PDL1 and BIRC5 (**Figures 8G, H**, **Table 4**).

**Table 4:** The miRNAs targeting STC2, BIRC5, and PDL1.

STC2	BIRC5	PDL1
hsa-miR-106a-5p	hsa-miR-15a-5p	hsa-let-7a-5p
hsa-miR-335-5p	hsa-miR-424-5p	hsa-let-7f-5p
hsa-miR-15b-5p	hsa-miR-218-5p	hsa-miR-15a-5p
hsa-miR-20b-5p	hsa-miR-550a-3p	hsa-miR-424-5p
hsa-miR-424-5p	hsa-miR-219a-5p	hsa-miR-182-5p
hsa-miR-15a-5p	hsa-miR-21-5p	hsa-miR-15b-5p
hsa-miR-708-5p	hsa-miR-33b-5p	hsa-miR-374a-5p
hsa-miR-125b-5p	hsa-miR-320d	hsa-miR-16-5p
hsa-miR-103a-3p	hsa-miR-223-3p	hsa-miR-195-5p
hsa-miR-130a-3p	hsa-miR-219a-1-3p	hsa-miR-17-5p
hsa-miR-16-5p	hsa-miR-16-5p	hsa-miR-155-5p
hsa-miR-374b-5p	hsa-miR-195-5p	hsa-miR-302c-3p
hsa-miR-106b-5p	hsa-miR-34a-5p	hsa-miR-106a-5p
hsa-miR-576-5p	hsa-miR-20b-5p	hsa-miR-15b-3p
hsa-miR-20a-5p	hsa-let-7g-5p	hsa-miR-106b-5p
hsa-miR-17-5p	hsa-miR-452-5p	hsa-let-7b-5p
hsa-miR-124-3p	hsa-miR-181b-5p	hsa-miR-20a-5p
hsa-miR-302a-3p	hsa-miR-129-2-3p	hsa-miR-107
hsa-miR-302d-3p	hsa-miR-1225-5p	hsa-miR-1246
hsa-miR-30e-5p	hsa-miR-671-5p	hsa-miR-1292-5p
hsa-miR-876-3p	hsa-miR-30c-2-3p	hsa-miR-24-3p
hsa-miR-887-3p	hsa-miR-106a-5p	hsa-miR-34a-5p
hsa-miR-545-5p	hsa-miR-30a-5p	hsa-miR-142-5p
hsa-miR-30a-5p	hsa-miR-17-5p	hsa-miR-9-3p
hsa-miR-34a-5p	hsa-miR-182-5p	hsa-miR-130a-3p
hsa-miR-301b-3p	hsa-miR-106b-5p	hsa-miR-150-3p
hsa-miR-454-3p	hsa-miR-194-5p	hsa-miR-3928-3p
hsa-miR-155-5p	hsa-miR-576-3p	hsa-miR-93-5p
hsa-miR-130b-3p	hsa-miR-203a-3p	hsa-miR-103a-3p
hsa-miR-181b-5p	hsa-miR-7-5p	hsa-miR-301b-3p
hsa-miR-132-3p	hsa-miR-30a-3p	hsa-miR-33a-5p
hsa-miR-181a-5p	hsa-miR-20a-5p	hsa-miR-30c-1-3p
hsa-miR-4491	hsa-miR-130b-3p	hsa-miR-23a-3p
hsa-miR-181d-5p	hsa-miR-124-3p	hsa-miR-320a-3p
hsa-miR-101-3p	hsa-miR-135a-5p	hsa-miR-20b-5p
hsa-miR-139-5p	hsa-miR-130a-3p	hsa-miR-320c
hsa-miR-24-3p	hsa-miR-148a-3p	hsa-miR-18a-5p
hsa-miR-27a-3p	hsa-miR-301a-3p	hsa-miR-363-3p
hsa-miR-27b-3p	hsa-miR-301b-3p	hsa-miR-2278
hsa-miR-449b-5p	hsa-miR-454-3p	hsa-miR-183-5p
hsa-let-7a-5p	hsa-miR-10a-5p	hsa-miR-25-3p
hsa-let-7c-5p	hsa-miR-10b-5p	hsa-miR-138-5p
hsa-let-7d-5p	hsa-miR-497-5p	hsa-miR-185-5p
hsa-let-7e-5p	hsa-miR-181a-5p	hsa-miR-301a-3p
hsa-let-7f-5p	hsa-miR-142-5p	hsa-miR-374b-5p
hsa-let-7g-5p	hsa-let-7b-5p	hsa-miR-30e-3p
hsa-let-7i-5p	hsa-miR-140-3p	hsa-miR-23c
hsa-miR-196a-5p	hsa-miR-148b-3p	hsa-miR-877-5p
hsa-miR-425-5p	hsa-miR-205-5p	hsa-miR-320b
hsa-miR-7-5p	hsa-miR-1180-3p	hsa-miR-23b-3p
hsa-miR-3140-3p	hsa-miR-181d-5p	hsa-miR-32-5p
hsa-miR-625-5p	hsa-miR-200a-3p	hsa-miR-7-5p
hsa-miR-18a-5p	hsa-miR-30d-3p	hsa-miR-3934-5p
hsa-miR-18b-5p	hsa-miR-30e-3p	hsa-miR-92a-3p
hsa-miR-671-5p	hsa-miR-320b	hsa-miR-18b-5p
hsa-miR-4306	hsa-miR-542-3p	hsa-miR-590-5p
hsa-miR-3177-3p	hsa-miR-93-5p	hsa-miR-92b-3p
hsa-miR-1827	hsa-let-7d-5p	hsa-miR-320d
hsa-miR-135b-3p	hsa-miR-15b-3p	hsa-miR-19a-3p
hsa-miR-378a-3p	hsa-miR-139-5p	hsa-miR-19b-3p
hsa-miR-28-5p	hsa-miR-141-3p	hsa-miR-5000-3p
hsa-miR-19b-3p	hsa-miR-27a-3p	hsa-miR-29c-3p
hsa-miR-182-5p	hsa-miR-877-5p	hsa-miR-30a-5p
hsa-miR-423-5p	hsa-miR-25-5p	hsa-miR-30d-5p
hsa-miR-147b-3p	hsa-let-7c-5p	hsa-miR-26a-5p
hsa-miR-193b-5p	hsa-miR-671-3p	hsa-miR-26b-5p
hsa-miR-191-5p	hsa-miR-4677-3p	hsa-miR-29b-3p
hsa-miR-92a-3p	hsa-miR-1307-5p	hsa-miR-194-5p
hsa-miR-15b-3p	hsa-miR-196a-5p	hsa-miR-29c-5p
hsa-miR-218-5p	hsa-miR-423-5p	hsa-miR-584-5p
hsa-miR-98-5p	hsa-miR-22-3p	hsa-miR-4677-3p
hsa-miR-19a-3p	hsa-miR-26b-5p	hsa-let-7d-5p
hsa-miR-449c-5p	hsa-miR-375-3p	hsa-let-7c-5p
hsa-miR-30a-3p	hsa-miR-149-5p	hsa-miR-148b-3p
hsa-miR-576-3p	hsa-miR-96-5p	hsa-let-7e-5p
hsa-miR-10a-5p	hsa-miR-151b	hsa-let-7i-5p
hsa-miR-152-3p	hsa-miR-101-3p	hsa-miR-221-3p
hsa-miR-183-5p	hsa-let-7i-5p	hsa-miR-302a-3p
hsa-miR-135b-5p	hsa-miR-484	hsa-miR-196a-5p
hsa-miR-96-5p	hsa-miR-152-3p	hsa-miR-148a-3p
hsa-miR-877-5p	hsa-miR-182-3p	hsa-miR-222-3p
hsa-miR-628-5p	hsa-miR-450a-5p	hsa-miR-335-3p
hsa-let-7b-5p	hsa-miR-99b-5p	hsa-miR-191-5p
hsa-miR-107	hsa-miR-1234-3p	hsa-miR-1271-5p
hsa-miR-195-5p	hsa-miR-3184-3p	hsa-miR-340-5p
hsa-miR-503-5p	hsa-miR-328-3p	hsa-miR-34b-5p
hsa-miR-411-3p	hsa-miR-320a-3p	hsa-miR-1-3p
hsa-miR-193a-3p	hsa-miR-203b-5p	
hsa-miR-193b-3p	hsa-miR-27b-3p	
hsa-miR-205-5p	hsa-miR-19a-3p	
hsa-miR-21-5p	hsa-miR-183-5p	
hsa-miR-497-5p	hsa-miR-103a-3p	
hsa-miR-125b-2-3p	hsa-miR-15b-5p	
hsa-miR-186-5p	hsa-miR-107	
hsa-miR-320a-3p	hsa-miR-148b-5p	
hsa-miR-4677-3p	hsa-miR-29a-3p	
hsa-miR-93-5p	hsa-miR-19b-3p	
hsa-miR-29c-3p	hsa-miR-423-3p	
hsa-miR-196b-5p	hsa-miR-486-3p	
hsa-miR-29a-3p	hsa-miR-29c-3p	
hsa-miR-641	hsa-miR-30d-5p	
hsa-miR-589-3p	hsa-miR-132-3p	
hsa-miR-429	hsa-miR-103b	
hsa-miR-1301-3p	hsa-miR-17-3p	
hsa-miR-320b	hsa-miR-760	
hsa-miR-577	hsa-miR-199a-3p	
hsa-miR-532-5p	hsa-miR-199b-3p	
hsa-miR-140-3p	hsa-let-7f-5p	
hsa-miR-148a-3p	hsa-miR-185-5p	
hsa-miR-30b-3p	hsa-let-7a-5p	
hsa-miR-194-5p	hsa-miR-210-3p	
hsa-miR-3909	hsa-miR-340-5p	
hsa-miR-4446-3p	hsa-miR-708-5p	
hsa-miR-200a-5p	hsa-miR-1-3p	
hsa-miR-30d-5p	hsa-miR-1343-3p	
hsa-miR-324-5p	hsa-miR-218-1-3p	
hsa-miR-489-3p	hsa-miR-26a-5p	
hsa-miR-203a-3p	hsa-miR-147a	
hsa-miR-26b-5p	hsa-miR-345-5p	
hsa-miR-33a-5p	hsa-miR-1296-5p	
hsa-miR-33b-5p	hsa-miR-335-5p	
hsa-miR-1266-5p	hsa-miR-128-3p	
hsa-miR-181c-5p		
hsa-miR-23a-3p		
hsa-miR-25-3p		
hsa-miR-326		
hsa-miR-92b-3p		
hsa-miR-30d-3p		
hsa-miR-197-3p		
hsa-miR-3620-3p		
hsa-miR-340-3p		
hsa-miR-4728-3p		
hsa-miR-769-5p		
hsa-let-7f-2-3p		
hsa-miR-516b-5p		
hsa-miR-185-5p		
hsa-miR-182-3p		
hsa-miR-340-5p		
hsa-miR-23b-3p		
hsa-miR-4709-5p		
hsa-miR-148a-5p		
hsa-miR-548e-3p		
hsa-miR-454-5p		
hsa-miR-4429		
hsa-miR-143-3p		
hsa-miR-30c-1-3p		
hsa-miR-1225-5p		
hsa-miR-3652		
hsa-miR-1910-5p		
hsa-miR-26a-5p		
hsa-miR-3184-5p		
hsa-miR-197-5p		
hsa-miR-378i		
hsa-let-7d-3p		
hsa-miR-103b		
hsa-miR-320d		
hsa-miR-455-5p		
hsa-miR-30e-3p		
hsa-miR-423-3p		
hsa-miR-574-5p		
hsa-miR-1271-5p		
hsa-miR-21-3p		
hsa-miR-27a-5p		
hsa-miR-147a		
hsa-miR-494-3p		
hsa-miR-941		
hsa-miR-138-5p		

## Discussion

Currently, nonsurgical therapeutic interventions are instrumental in the management of HCC, as the majority of patients present with advanced disease stages that preclude surgical intervention (2). As immunotherapy continues to evolve, the role of immunotherapies in the management of HCC has become increasingly pivotal, exerting a profound influence on patient prognosis. In this study, we constructed a prognostic and immunotherapy efficacy prediction model SIR-PS based on two distinct HCC molecular subtypes. This model consists of four genes: *STC2*, *BIRC5*, *EPO*, and *GLP1R*. Using a group of genes to build the prognostic model was successfully used in some solid tumors, such as breast cancer (36, 37). But to our knowledge, this is the first-time using SIR-PS to predict the prognosis and give suggestion of immune therapy in HCC.

Further validation on HCC cell lines revealed distinct RNA or protein expression levels of *STC2* and *BIRC5* in different malignant HCC cell lines, which were correspondence with these cells’ malignances. STC2 has been revealed a marked increased expression in HCC tissues compared to normal tissues (38). Additionally, *STC2* has also been implicated in promoting tumor cell invasion and metastasis while concurrently inhibiting apoptosis in numerous tumor types (39). This heightened expression was positively correlated with an adverse patient prognosis, et al. which was consistent with our results. There was also been reported a significant overexpression of *BIRC5* in HCC tissues, contrast to its near undetectability in tissues affected by cirrhosis (40). The expression of *BIRC5* appears to be correlated with the metastatic potential of HCC, which is aligns with the findings of this study.

However, same trends didn’t been observed on *EPO* and *GLP1R* in different HCC cells. In this study, we found that *EPO and GLP1R* could promote HCC development and coincident with worse prognosis by bioinformation data and the validated results on HCC cohorts. However, the validation in different HCC cell lines didn’t show obvious relationship with their corresponding malignances. The protein levels of *EPO* and *GLP1R* were even no statistical differences. Further exploration on the expression of these genes in HCC cell cohorts was taken out by spatial transcriptome analysis. Differ from STC2 and BIRC5 which were mainly expressed in liver cancer cells, *EPO* and *GLP1R* did not exhibit specific expression in a certain cell type, which could potentially be attributed to the fact that *EPO* and *GLP1R* may not predominantly expressed in HCC cell lines.

Analysis of the mutational landscape of genes between low-risk and high-risk groups of HCC revealed significant differences in TP53 and CTNNB1. *TP53* mutations are correlated with an unfavorable prognosis in HCC patients, and are predictive of potential responsiveness to immunotherapy (41). In various cell lines, TP53 mutations or knockdown lead to increased PDL1 expression (42, 43). Conversely, *CTNNB1* mutations, while indicative of a favorable prognosis, are linked to reduced efficacy of immunotherapy in HCC patients (44, 45). And patients with CTNNB1 mutations exhibit lower PDL1 expression (46, 47). Therefore, TP53 and CTNNB1 may influence the efficacy of immunotherapy by affecting PDL1 expression. These findings supported SIR-PS as the predictive model for HCC prognosis and immunotherapy efficacy. Meanwhile, validation on external HCC cohorts and gathering the corresponding clinical characteristics proofed SIR-PS as an apt prognostic model for HCC patients, demonstrating robust predictive accuracy in forecasting clinical outcomes. Patients categorized in the high-risk group by SIR-PS exhibited significantly adverse prognosis.

Moreover, microenvironment analysis showed this model could serves as an excellent and dependable tool for the prediction of treatment responses to immunotherapy. CD8+ T cells were the primary immune cells that exert anti-tumor effects (48). The expression of PDL1 on tumor cells often led to the exhaustion or reduced function of CD8+ T cells (49, 50). The mechanism of anti-PD-1 therapy is to restore the function of exhausted CD8 T cells and promote their proliferation (51, 52). In this study, a higher infiltration level of CD8+ T cell was observed within the high risk group. High CD8 T cells pave the way for anti-PD-1 therapy to restore those exhausted T cell function and finally killed the tumor cells. Meanwhile, we also detected the immune checkpoint gene expressions between risk groups which revealed that in contrast to the low-risk group, the high-risk group displayed elevated expression levels for most of these genes. This should be a direct clue for anti-PD-1/PDL1 efficiency.

In order to explain the potential mechanisms of prognostic models on the efficacy of immunotherapy, especially on the expression of PDL1, we further explored STC2 and BIRC5 form endogenous competitive RNAs with PDL1 through multiple miRNAs, which affect the expression of PDL1. STC2 and PDL1 mRNAs can compete with each other for binding to miR-17-5p, miR-33a, miR-34a, miR-138-5p, miR-140, miR-152, miR-155, miR-197, miR-200, and miR-424 (53–68). Additionally, BIRC5 and PDL1 mRNAs also compete with each other for binding to miR-17-5p, miR-34a, miR-140, miR-142-5p, miR-152, miR-200, and miR-424 (53, 54, 56, 58, 59, 63–66, 69, 70). Consequently, an increase in the expression level of one mRNA enhances its competitive binding with miRNAs, which in turn can lead to an increase in the expression level of another mRNA to a certain extent. The elevated expression of STC2 and BIRC5, can promote the binding with those competed miRNA of PDL1, which in turn upregulated the PDL1 expression. Simultaneously, the activation of the PI3K/AKT pathway is known to promote PD-L1 expression (71–73). Li and Zhu et al.’s research demonstrates that STC2 can facilitate the activation of the PI3K/AKT pathway (74, 75). Additionally, Shang et al.’s research have shown that BIRC5 expression is regulated by the PI3K/AKT pathway (76). Thus, elevated BIRC5 expression may serve as an indicator of PI3K/AKT pathway activation.

Further validation of the prognostic predictive ability of SIR-PS on HCC tissues of our own center were taken out, and the consistent results were collected. In addition, we creatively combined SIR-PS with IHC, which is more extensively utilized and offers greater convenience in clinical application in comparison to RNA-Seq technology. HCC tissue chips were performed for IHC staining and the results were scored. Utilizing these scores, we employed the SIR-PS to calculate individual patient risk scores, thereby evaluating the clinical utility of it. The SIR-PS exhibited a high degree of accuracy in prognostically assessing the 1 and 3 year survival for the HCC tissue chips’ patients, with the low-risk group exhibiting a markedly more favorable prognosis than the high-risk group. In comparison to other immunohistochemical indicators, such as *GPC3* and *CK19*, the SIR-PS demonstrates superior predictive capabilities. This study has to some extent filled the gap in clinical pathological work that lacks specific IHC prognostic indicators for HCC. However, there are still limitation and deficiency in our study. Firstly, due to the lack of immune therapy results in tissue chips, we were unable to validate the predictive ability of this model for immune therapy efficacy in tissue chips through IHC. Secondly, although the datasets we included cover a wide range of ethnicities, they are still not comprehensive. Finally, as the datasets only include samples from patients who can undergo surgery, the applicability to samples from patients who cannot undergo surgery is uncertain, especially in clinical pathology work, where liver biopsy samples from non-resectable patients may not be applicable.

Taken together, the present investigation identified a novel prognostic model (SIR-PS) based on the KEGG pathway and focused on immune related genes. This model demonstrates potential as an effective tool for predicting prognosis of HCC and for assessing the efficacy of immunotherapeutic interventions. Utilizing the SIR-PS to calculate the risk score of each patient with HCC has showed a favorable efficacy in the 1 and 3 year survival rate prognostication. Given the absence of specific biomarkers for the prognostic evaluation of HCC in clinical, combination of SIR-PS with IHC promoted the clinical application of prognostic models and broadening the approach of prognostic models from databases to clinical practice.

## Data Availability

Publicly available datasets were analyzed in this study. This data can be found here: TCGA LIHC (https://portal.gdc.cancer.gov/);LIRI-JP of ICGC (https://dcc.icgc.org/);ImmPort (https://immport.niaid.nih.gov/);GSE54236 and GSE202069(https://www.ncbi.nlm.nih.gov/geo/).
