# Supplementary material for: An immune-related signature based on molecular subtypes for predicting the prognosis and immunotherapy efficacy of hepatocellular carcinoma
Source: Front Immunol. 2025 May 19;16:1481366. doi: 10.3389/fimmu.2025.1481366 (PMC12127308; doi:10.3389/fimmu.2025.1481366)
Supplement: Supplementary file 1 [file DataSheet1.docx]

***Supplementary Material***

# Supplementary Figures and Tables

## Supplementary Figures


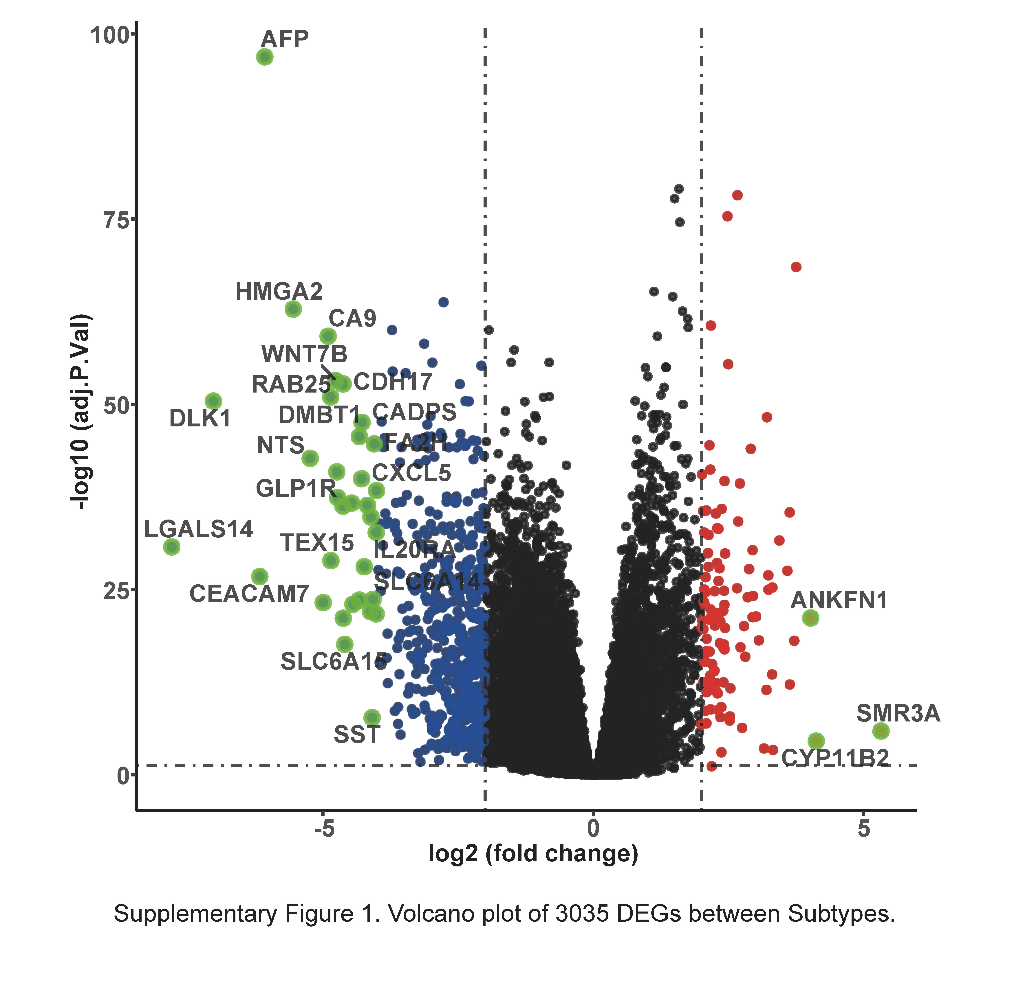


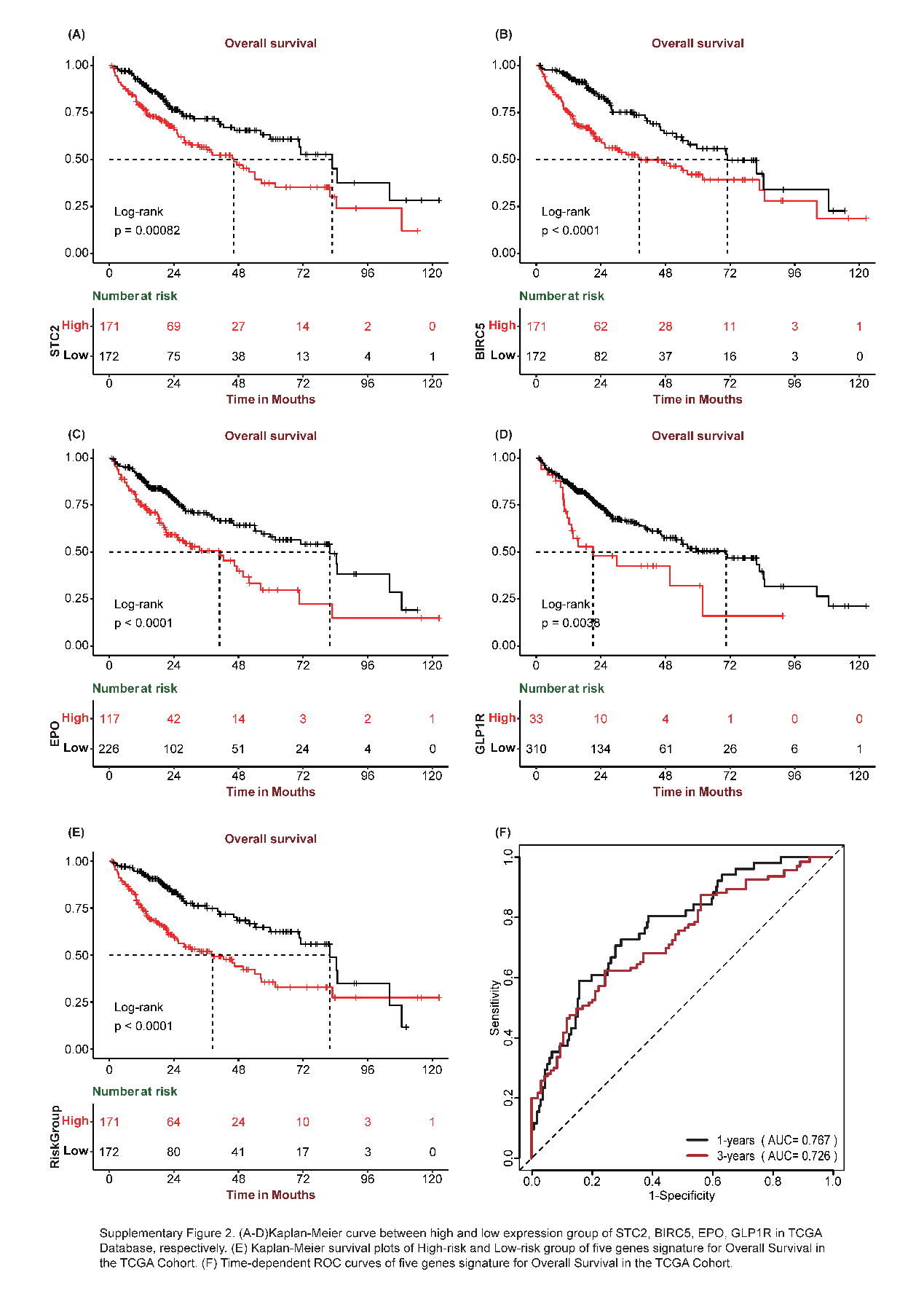


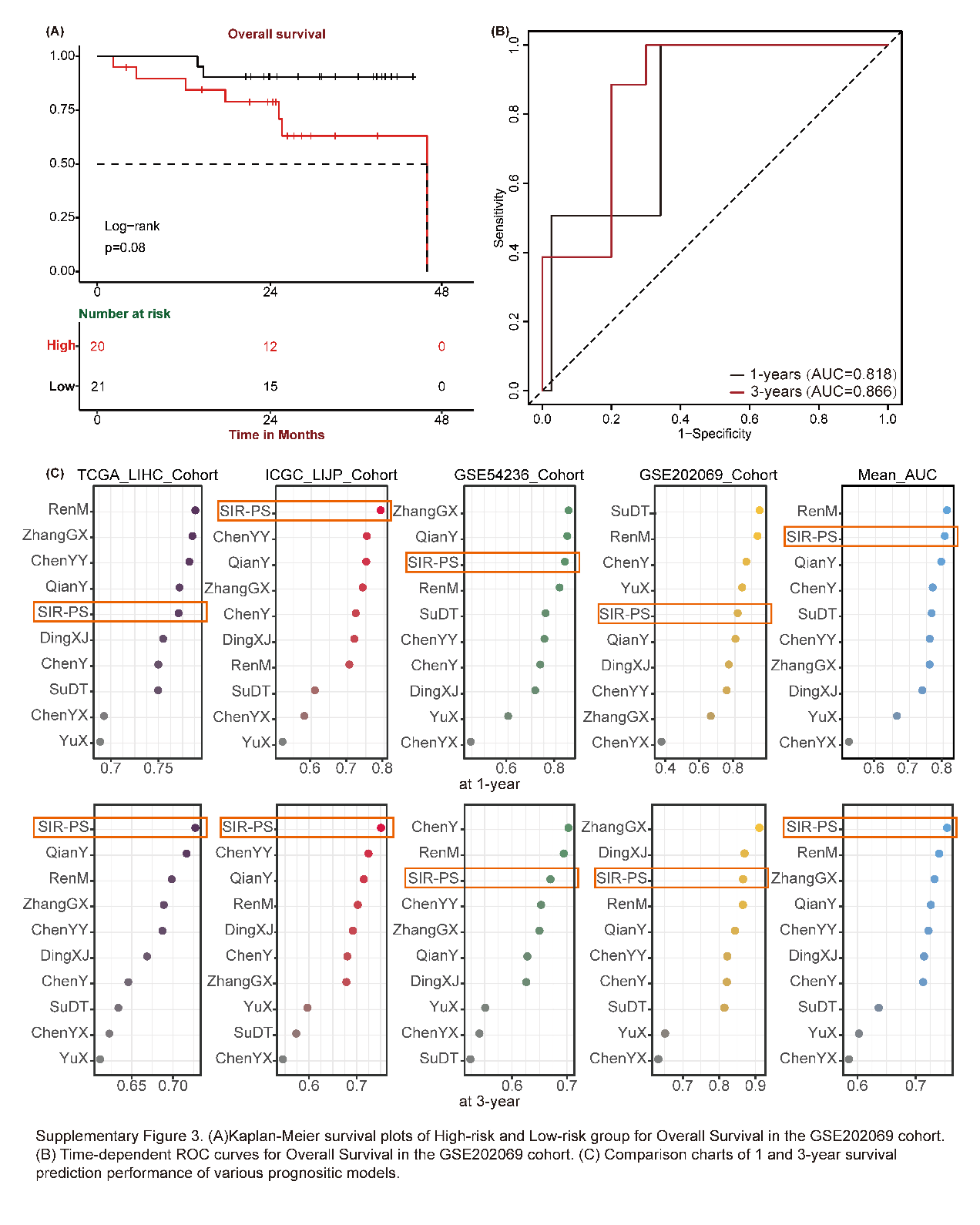


## Supplementary Tables

**Supplementary Table 1** Subtype of TCGA-LIHC cohort patients.

| Subtype | TCGA sample id |
| --- | --- |
| Sub1 | TCGA.DD.A4NG.01A, TCGA.BC.A10Y.01A, TCGA.K7.AAU7.01A, TCGA.BC.A10W.01A, TCGA.DD.AAD3.01A, TCGA.DD.A1EI.01A, TCGA.GJ.A6C0.01A, TCGA.CC.5258.01A, TCGA.DD.AADP.01A, TCGA.DD.AACW.01A, TCGA.UB.A7MF.01A, TCGA.CC.A8HV.01A, TCGA.DD.AADB.01A, TCGA.DD.A1EA.01A, TCGA.DD.A113.01A, TCGA.CC.A7IE.01A, TCGA.XR.A8TD.01A, TCGA.CC.A9FW.01A, TCGA.G3.A5SJ.01A, TCGA.G3.A7M9.01A, TCGA.2Y.A9GS.01A, TCGA.ED.A7PX.01A, TCGA.DD.AADC.01A, TCGA.DD.AAVZ.01A, TCGA.CC.A3MA.01A, TCGA.2Y.A9GY.01A, TCGA.ED.A627.01A, TCGA.2Y.A9GW.01A, TCGA.DD.AADO.01A, TCGA.DD.A1EH.01A, TCGA.G3.AAV7.01A, TCGA.DD.AACA.01A, TCGA.FV.A3R3.01A, TCGA.DD.AACC.01A, TCGA.BC.A3KG.01A, TCGA.CC.A7IK.01A, TCGA.UB.A7MD.01A, TCGA.DD.A4NS.01A, TCGA.CC.A9FU.01A, TCGA.DD.AACP.01A, TCGA.2Y.A9H8.01A, TCGA.FV.A3I1.01A, TCGA.2Y.A9H7.01A, TCGA.ZP.A9CZ.01A, TCGA.DD.AAE1.01A, TCGA.GJ.A3OU.01A, TCGA.DD.AAVS.01A, TCGA.CC.A8HS.01A, TCGA.FV.A3I0.01A, TCGA.XR.A8TE.01A, TCGA.CC.A3MB.01A, TCGA.MI.A75I.01A, TCGA.DD.AACZ.01A, TCGA.CC.A8HU.01A, TCGA.RC.A6M5.01A, TCGA.BC.4073.01B, TCGA.UB.AA0U.01A, TCGA.G3.A5SI.01A, TCGA.FV.A4ZQ.01A, TCGA.DD.A4ND.01A, TCGA.ED.A8O5.01A, TCGA.UB.A7ME.01A, TCGA.CC.5264.01A, TCGA.DD.AADA.01A, TCGA.DD.AACS.01A, TCGA.DD.AAE0.01A, TCGA.DD.AAVQ.01A, TCGA.RC.A7SH.01A, TCGA.BC.A10Q.01A, TCGA.DD.A1EE.01A, TCGA.FV.A23B.01A, TCGA.CC.A1HT.01A, TCGA.EP.A2KB.01A, TCGA.DD.A4NN.01A, TCGA.DD.AAD1.01A, TCGA.DD.AAVW.01A, TCGA.DD.AACN.01A, TCGA.DD.AADD.01A, TCGA.DD.AACI.01A, TCGA.ZP.A9D1.01A, TCGA.DD.A4NR.01A, TCGA.RG.A7D4.01A, TCGA.BC.A217.01A, TCGA.RC.A6M3.01A, TCGA.DD.A4NQ.01A, TCGA.CC.A9FV.01A, TCGA.UB.A7MC.01A, TCGA.MR.A8JO.01A, TCGA.CC.A123.01A, TCGA.YA.A8S7.01A, TCGA.2Y.A9H2.01A, TCGA.5C.A9VG.01A, TCGA.CC.A3M9.01A, TCGA.EP.A2KA.01A, TCGA.DD.AADW.01A, TCGA.DD.AAED.01A, TCGA.ZP.A9D2.01A, TCGA.G3.A25Y.01A, TCGA.K7.A5RG.01A, TCGA.EP.A3RK.01A, TCGA.CC.A7IJ.01A, TCGA.ED.A7XP.01A, TCGA.ED.A5KG.01A, TCGA.ED.A459.01A, TCGA.2Y.A9GX.01A, TCGA.QA.A7B7.01A, TCGA.DD.AACL.01A, TCGA.DD.A1EF.01A, TCGA.G3.A25T.01A, TCGA.FV.A4ZP.01A, TCGA.WX.AA44.01A, TCGA.CC.A7IG.01A, TCGA.G3.A25X.01A, TCGA.BC.4072.01B, TCGA.XR.A8TC.01A, TCGA.DD.AADI.01A, TCGA.BC.A8YO.01A, TCGA.DD.A39V.01A, TCGA.DD.A1EC.01A, TCGA.KR.A7K7.01A, TCGA.DD.AAVV.01A, TCGA.DD.AADN.01A, TCGA.RC.A7S9.01A, TCGA.BC.A112.01A, TCGA.2Y.A9H0.01A, TCGA.KR.A7K2.01A, TCGA.ED.A66Y.01A, TCGA.CC.A8HT.01A, TCGA.DD.AADY.01A, TCGA.BC.A216.01A, TCGA.G3.AAV6.01A, TCGA.DD.A1EG.01A, TCGA.PD.A5DF.01A, TCGA.DD.A3A9.01A, TCGA.DD.A118.01A, TCGA.5C.A9VH.01A, TCGA.DD.A3A6.01A, TCGA.CC.A5UD.01A, TCGA.DD.A4NH.01A, TCGA.BW.A5NO.01A, TCGA.XR.A8TG.01A, TCGA.DD.A1EJ.01A, TCGA.2Y.A9H3.01A, TCGA.DD.AAD5.01A, TCGA.BD.A3EP.01A, TCGA.CC.5260.01A, TCGA.WQ.A9G7.01A, TCGA.FV.A2QR.01A, TCGA.DD.AA3A.01A, TCGA.CC.5263.01A, TCGA.BC.A10U.01A, TCGA.5R.AA1D.01A, TCGA.ED.A82E.01A, TCGA.BC.A69H.01A, TCGA.CC.5261.01A, TCGA.CC.A7II.01A, TCGA.CC.A3MC.01A, TCGA.2V.A95S.01A, TCGA.ED.A66X.01A, TCGA.DD.AACH.01A, TCGA.CC.5262.01A, TCGA.RC.A6M6.01A, TCGA.CC.A5UE.01A, TCGA.DD.AACF.01A, TCGA.BW.A5NP.01A, TCGA.UB.A7MA.01A, TCGA.DD.A4NA.01A, TCGA.5C.AAPD.01A, TCGA.KR.A7K8.01A, TCGA.DD.A4NB.01A, TCGA.DD.A11C.01A, TCGA.WJ.A86L.01A, TCGA.ED.A8O6.01A, TCGA.BW.A5NQ.01A, TCGA.DD.A1EL.01A, TCGA.DD.A4NJ.01A, TCGA.DD.AACG.01A, TCGA.DD.AAC8.01A, TCGA.DD.AACB.01A, TCGA.G3.A7M6.01A, TCGA.ED.A97K.01A, TCGA.CC.A5UC.01A, TCGA.DD.A39Y.01A, TCGA.DD.A114.01A, TCGA.2Y.A9H5.01A |
| Sub2 | TCGA.G3.AAV4.01A, TCGA.2Y.A9H1.01A, TCGA.DD.AACV.01A, TCGA.DD.AAC9.01A, TCGA.DD.AACT.01A, TCGA.ZS.A9CD.01A, TCGA.WX.AA46.01A, TCGA.DD.AADQ.01A, TCGA.5R.AA1C.01A, TCGA.2Y.A9H9.01A, TCGA.DD.AAD2.01A, TCGA.DD.AACY.01A, TCGA.CC.A7IF.01A, TCGA.DD.AACD.01A, TCGA.ED.A4XI.01A, TCGA.DD.AAE2.01A, TCGA.EP.A2KC.01A, TCGA.DD.AADK.01A, TCGA.KR.A7K0.01A, TCGA.G3.AAV2.01A, TCGA.G3.A25S.01A, TCGA.DD.AADR.01A, TCGA.DD.AAE3.01A, TCGA.DD.AAVU.01A, TCGA.BC.A110.01A, TCGA.RC.A7SB.01A, TCGA.2Y.A9GT.01A, TCGA.2Y.A9HA.01A, TCGA.FV.A2QQ.01A, TCGA.DD.AAEH.01A, TCGA.DD.AADM.01A, TCGA.G3.A3CK.01A, TCGA.EP.A26S.01A, TCGA.DD.A11A.01A, TCGA.DD.AAE6.01A, TCGA.DD.A73B.01A, TCGA.EP.A3JL.01A, TCGA.DD.AAD0.01A, TCGA.2Y.A9GZ.01A, TCGA.DD.AACO.01A, TCGA.LG.A6GG.01A, TCGA.ED.A7PZ.01A, TCGA.DD.A4NV.01A, TCGA.DD.A73G.01A, TCGA.G3.AAV3.01A, TCGA.G3.A25U.01A, TCGA.BC.A5W4.01A, TCGA.EP.A12J.01A, TCGA.2Y.A9HB.01A, TCGA.G3.A5SK.01A, TCGA.BC.A69I.01A, TCGA.BC.A10R.01A, TCGA.BC.A10X.01A, TCGA.BC.A10S.01A, TCGA.DD.AAVX.01A, TCGA.G3.A3CJ.01A, TCGA.DD.A115.01A, TCGA.ZP.A9CY.01A, TCGA.HP.A5MZ.01A, TCGA.DD.AACK.01A, TCGA.DD.A11B.01A, TCGA.ZP.A9D4.01A, TCGA.HP.A5N0.01A, TCGA.ES.A2HS.01A, TCGA.G3.A3CG.01A, TCGA.DD.AAVR.01A, TCGA.DD.A73D.01A, TCGA.DD.A4NF.01A, TCGA.DD.A3A2.01A, TCGA.K7.A5RF.01A, TCGA.DD.A116.01A, TCGA.DD.A4NI.01A, TCGA.BC.A10T.01A, TCGA.DD.AADJ.01A, TCGA.DD.AACE.01A, TCGA.FV.A3R2.01A, TCGA.DD.AAE7.01A, TCGA.4R.AA8I.01A, TCGA.FV.A496.01A, TCGA.DD.A39X.01A, TCGA.DD.AADS.01A, TCGA.DD.AAE9.01A, TCGA.G3.AAUZ.01A, TCGA.WX.AA47.01A, TCGA.NI.A4U2.01A, TCGA.UB.A7MB.01A, TCGA.DD.AAVP.01A, TCGA.5R.AAAM.01A, TCGA.DD.A3A4.01A, TCGA.DD.AAEG.01A, TCGA.DD.A39W.01A, TCGA.DD.A39Z.01A, TCGA.BC.A10Z.01A, TCGA.WQ.AB4B.01A, TCGA.LG.A9QD.01A, TCGA.DD.AADV.01A, TCGA.G3.A3CH.01A, TCGA.G3.AAV1.01A, TCGA.ZS.A9CG.01A, TCGA.DD.AAW3.01A, TCGA.ED.A7PY.01A, TCGA.G3.A3CI.01A, TCGA.MI.A75H.01A, TCGA.G3.AAV5.01A, TCGA.DD.A1ED.01A, TCGA.DD.A73E.01A, TCGA.MR.A520.01A, TCGA.DD.A4NE.01A, TCGA.ZP.A9D0.01A, TCGA.BC.A3KF.01A, TCGA.O8.A75V.01A, TCGA.DD.AACQ.01A, TCGA.DD.A1EB.01A, TCGA.LG.A9QC.01A, TCGA.CC.A7IL.01A, TCGA.DD.AADU.01A, TCGA.RC.A7SF.01A, TCGA.RC.A7SK.01A, TCGA.DD.AAD8.01A, TCGA.DD.AAEE.01A, TCGA.DD.A119.01A, TCGA.DD.A73F.01A, TCGA.DD.A4NP.01A, TCGA.DD.AAEK.01A, TCGA.DD.A11D.01A, TCGA.2Y.A9H4.01A, TCGA.DD.A4NL.01A, TCGA.3K.AAZ8.01A, TCGA.MI.A75G.01A, TCGA.DD.A3A3.01A, TCGA.CC.A7IH.01A, TCGA.K7.A6G5.01A, TCGA.DD.AACU.01A, TCGA.CC.5259.01A, TCGA.UB.AA0V.01A, TCGA.GJ.A9DB.01A, TCGA.DD.A4NK.01A, TCGA.2Y.A9H6.01A, TCGA.BD.A2L6.01A, TCGA.G3.A5SM.01A, TCGA.DD.AAW2.01A, TCGA.FV.A495.01A, TCGA.ZS.A9CE.01A, TCGA.G3.A7M8.01A, TCGA.DD.A3A1.01A, TCGA.MI.A75E.01A, TCGA.DD.AAEA.01A, TCGA.ZS.A9CF.01A, TCGA.G3.A25V.01A, TCGA.CC.A9FS.01A, TCGA.DD.A3A7.01A, TCGA.G3.AAV0.01A, TCGA.DD.AAE4.01A, TCGA.DD.A3A8.01A, TCGA.G3.A7M5.01A, TCGA.G3.A6UC.01A, TCGA.BD.A3ER.01A, TCGA.DD.A3A5.01A, TCGA.DD.AADG.01A, TCGA.DD.AAW0.01A, TCGA.DD.AACJ.01A, TCGA.DD.AADL.01A, TCGA.DD.A4NO.01A, TCGA.G3.A7M7.01A, TCGA.ES.A2HT.01A, TCGA.DD.AACX.01A, TCGA.2Y.A9GV.01A, TCGA.G3.A25Z.01A, TCGA.MI.A75C.01A, TCGA.ZP.A9CV.01A, TCGA.T1.A6J8.01A, TCGA.DD.A1EK.01A, TCGA.DD.AAD6.01A, TCGA.DD.AAEB.01A, TCGA.2Y.A9GU.01A, TCGA.DD.A73A.01A, TCGA.RC.A6M4.01A, TCGA.DD.AAVY.01A, TCGA.G3.A5SL.01A, TCGA.ED.A7XO.01A, TCGA.DD.A73C.01A, TCGA.DD.AAW1.01A, TCGA.XR.A8TF.01A, TCGA.NI.A8LF.01A, TCGA.DD.AADF.01A, TCGA.DD.AAEI.01A |

Supplementary Table 2. 67 Immune-related Differentially Expressed Genes among HCC Subtypes with significant prognostic potential

| PLXNA1 | NTS | GLP1R | AZGP1 | CXCL5 | ESR1 | IL20RA |
| --- | --- | --- | --- | --- | --- | --- |
| CSF3R | IKBKE | CMTM3 | PLAUR | IL12A | PLXNA3 | CTSE |
| IL17RD | SEMA3A | OXTR | IL27 | MMP9 | KLKB1 | TGFB1 |
| NR0B1 | AQP9 | AR | CHGA | CCL26 | TNFRSF11A | CD79A |
| MASP1 | S100A6 | TNFRSF21 | CCL16 | MASP2 | KCNH2 | PROC |
| IL11 | PTGER4 | TFR2 | NR1I2 | BIRC5 | TNFSF4 | NDRG1 |
| KNG1 | LIF | ADRB2 | HRG | GHR | IGF1 | RNASE2 |
| SEMA6A | SLIT1 | STC2 | NDP | CALCR | CXCL1 | FGF11 |
| GDF10 | FGF9 | PTX3 | GAST | NRG1 | LEFTY2 | CLDN4 |
| GAL | GLP2R | EPO | DEFA5 |  |  |  |

Supplementary Table 3 Summary descriptives table of different indicators groups in the tissue chips cohort.

|  | STC2 | | | BIRC5 | | | EPO | | | GLP1R | | |
| --- | --- | --- | --- | --- | --- | --- | --- | --- | --- | --- | --- | --- |
|  | Low | High | p.overall | Low | High | p.overall | Low | High | p.overall | Low | High | p.overall |
| N: | 30 (37.5%) | 50 (62.5%) |  | 31 (38.8%) | 46 (61.2%) |  | 47 (58.8%) | 33 (41.2%) |  | 10 (12.5%) | 70 (87.5%) |  |
| Gender: |  |  | 0.773 |  |  | 0.688 |  |  | 0.609 |  |  | 1 |
| Female | 5 (16.7%) | 11 (22.0%) |  | 5 (16.1%) | 11 (22.4%) |  | 8 (17.0%) | 8 (24.2%) |  | 2 (20.0%) | 14 (20.0%) |  |
| Male | 25 (83.3%) | 39 (78.0%) |  | 26 (83.9%) | 38 (77.6%) |  | 39 (83.0%) | 25 (75.8%) |  | 8 (80.0%) | 56 (80.0%) |  |
| Age: |  |  | 1 |  |  | 0.273 |  |  | 0.851 |  |  | 0.513 |
| <=50 | 15 (50.0%) | 24 (48.0%) |  | 18 (58.1%) | 21 (42.9%) |  | 22 (46.8%) | 17 (51.5%) |  | 6 (60.0%) | 33 (47.1%) |  |
| >50 | 15 (50.0%) | 26 (52.0%) |  | 13 (41.9%) | 28 (57.1%) |  | 25 (53.2%) | 16 (48.5%) |  | 4 (40.0%) | 37 (52.9%) |  |
| ALT: |  |  | 0.839 |  |  | 1 |  |  | 0.475 |  |  | 0.694 |
| <=41 | 22 (73.3%) | 39 (78.0%) |  | 24 (77.4%) | 37 (75.5%) |  | 34 (72.3%) | 27 (81.8%) |  | 7 (70.0%) | 54 (77.1%) |  |
| >41 | 8 (26.7%) | 11 (22.0%) |  | 7 (22.6%) | 12 (24.5%) |  | 13 (27.7%) | 6 (18.2%) |  | 3 (30.0%) | 16 (22.9%) |  |
| AST: |  |  | 0.801 |  |  | 0.057 |  |  | 1 |  |  | 0.715 |
| <=40 | 22 (73.3%) | 34 (68.0%) |  | 26 (83.9%) | 30 (61.2%) |  | 33 (70.2%) | 23 (69.7%) |  | 8 (80.0%) | 48 (68.6%) |  |
| >40 | 8 (26.7%) | 16 (32.0%) |  | 5 (16.1%) | 19 (38.8%) |  | 14 (29.8%) | 10 (30.3%) |  | 2 (20.0%) | 22 (31.4%) |  |
| AFP: |  |  | 0.651 |  |  | 0.854 |  |  | 0.654 |  |  | 0.708 |
| <=20 | 6 (20.7%) | 14 (28.0%) |  | 7 (22.6%) | 13 (27.1%) |  | 13 (28.3%) | 7 (21.2%) |  | 3 (30.0%) | 17 (24.6%) |  |
| >20 | 23 (79.3%) | 36 (72.0%) |  | 24 (77.4%) | 35 (72.9%) |  | 33 (71.7%) | 26 (78.8%) |  | 7 (70.0%) | 52 (75.4%) |  |
| Child-Pugh: |  |  | 0.291 |  |  | 0.154 |  |  | 0.301 |  |  | 1 |
| A | 30 (100%) | 46 (92.0%) |  | 31 (100%) | 45 (91.8%) |  | 46 (97.9%) | 30 (90.9%) |  | 10 (100%) | 66 (94.3%) |  |
| B | 0 (0.00%) | 4 (8.00%) |  | 0 (0.00%) | 4 (8.16%) |  | 1 (2.13%) | 3 (9.09%) |  | 0 (0.00%) | 4 (5.71%) |  |
| Cirrhosis: |  |  | 0.388 |  |  | 1 |  |  | 0.707 |  |  | 0.28 |
| No | 12 (40.0%) | 14 (28.0%) |  | 10 (32.3%) | 16 (32.7%) |  | 14 (29.8%) | 12 (36.4%) |  | 5 (50.0%) | 21 (30.0%) |  |
| Yes | 18 (60.0%) | 36 (72.0%) |  | 21 (67.7%) | 33 (67.3%) |  | 33 (70.2%) | 21 (63.6%) |  | 5 (50.0%) | 49 (70.0%) |  |
| Tumor number: |  |  | 0.394 |  |  | 0.393 |  |  | 0.264 |  |  | 0.278 |
| 1 | 20 (66.7%) | 39 (78.0%) |  | 25 (80.6%) | 34 (69.4%) |  | 32 (68.1%) | 27 (81.8%) |  | 9 (90.0%) | 50 (71.4%) |  |
| >1 | 10 (33.3%) | 11 (22.0%) |  | 6 (19.4%) | 15 (30.6%) |  | 15 (31.9%) | 6 (18.2%) |  | 1 (10.0%) | 20 (28.6%) |  |
| Tumor size: |  |  | 0.678 |  |  | 0.237 |  |  | 1 |  |  | 0.733 |
| <=5cm | 13 (43.3%) | 18 (36.0%) |  | 9 (29.0%) | 22 (44.9%) |  | 18 (38.3%) | 13 (39.4%) |  | 3 (30.0%) | 28 (40.0%) |  |
| >5cm | 17 (56.7%) | 32 (64.0%) |  | 22 (71.0%) | 27 (55.1%) |  | 29 (61.7%) | 20 (60.6%) |  | 7 (70.0%) | 42 (60.0%) |  |
| Vascular invasion: |  |  | 0.649 |  |  | 0.964 |  |  | 1 |  |  | 0.683 |
| No | 26 (86.7%) | 40 (80.0%) |  | 25 (80.6%) | 41 (83.7%) |  | 39 (83.0%) | 27 (81.8%) |  | 9 (90.0%) | 57 (81.4%) |  |
| Yes | 4 (13.3%) | 10 (20.0%) |  | 6 (19.4%) | 8 (16.3%) |  | 8 (17.0%) | 6 (18.2%) |  | 1 (10.0%) | 13 (18.6%) |  |
| Differentation: |  |  | 1 |  |  | 0.835 |  |  | 0.389 |  |  | 0.011 |
| Moderate or High | 20 (66.7%) | 34 (68.0%) |  | 20 (64.5%) | 34 (69.4%) |  | 34 (72.3%) | 20 (60.6%) |  | 3 (30.0%) | 51 (72.9%) |  |
| Low or Moderately low | 10 (33.3%) | 16 (32.0%) |  | 11 (35.5%) | 15 (30.6%) |  | 13 (27.7%) | 13 (39.4%) |  | 7 (70.0%) | 19 (27.1%) |  |
| BCLC.stage: |  |  | 1 |  |  | 0.866 |  |  | 0.617 |  |  | 0.481 |
| A | 19 (63.3%) | 33 (66.0%) |  | 21 (67.7%) | 31 (63.3%) |  | 29 (61.7%) | 23 (69.7%) |  | 8 (80.0%) | 44 (62.9%) |  |
| B or C | 11 (36.7%) | 17 (34.0%) |  | 10 (32.3%) | 18 (36.7%) |  | 18 (38.3%) | 10 (30.3%) |  | 2 (20.0%) | 26 (37.1%) |  |
| TNM.stage: |  |  | 1 |  |  | 1 |  |  | 0.857 |  |  | 0.437 |
| 1 or 2 | 23 (76.7%) | 38 (76.0%) |  | 24 (77.4%) | 37 (75.5%) |  | 35 (74.5%) | 26 (78.8%) |  | 9 (90.0%) | 52 (74.3%) |  |
| 3 or 4 | 7 (23.3%) | 12 (24.0%) |  | 7 (22.6%) | 12 (24.5%) |  | 12 (25.5%) | 7 (21.2%) |  | 1 (10.0%) | 18 (25.7%) |  |
